# Marginal specificity in protein interactions constrains evolution of a paralogous family

**DOI:** 10.1073/pnas.2221163120

**Published:** 2023-04-25

**Authors:** Dia A. Ghose, Kaitlyn E. Przydzial, Emily M. Mahoney, Amy E. Keating, Michael T. Laub

**Affiliations:** ^a^Department of Biology, Massachusetts Institute of Technology, Cambridge, MA 02139; ^b^Department of Biological Engineering, Massachusetts Institute of Technology, Cambridge, MA 02139; ^c^Koch Center for Integrative Cancer Research, Massachusetts Institute of Technology, Cambridge, MA 02139; ^d^HHMI, Massachusetts Institute of Technology, Cambridge, MA 02139

**Keywords:** protein evolution, signal transduction, paralogous proteins, gene duplication, protein-protein interactions

## Abstract

The evolution of novel functions in biology relies heavily on gene duplication and divergence, creating large paralogous protein families. Selective pressure to avoid detrimental cross-talk often results in paralogs that exhibit exquisite specificity for their interaction partners. But how robust or sensitive is this specificity to mutation? Here, using deep mutational scanning, we demonstrate that a paralogous family of bacterial signaling proteins exhibits marginal specificity, such that many individual substitutions give rise to substantial cross-talk between normally insulated pathways. Our results indicate that sequence space is locally crowded despite overall sparseness, and we provide evidence that this crowding has constrained the evolution of bacterial signaling proteins. These findings underscore how evolution selects for “good enough” rather than optimized phenotypes, leading to restrictions on the subsequent evolution of paralogs.

## Significance

Large paralogous protein families are found throughout biology, the product of extensive gene duplication. To execute different functions inside cells, paralogs often acquire different specificities, interacting with desired, cognate partners and avoiding cross-talk with noncognate proteins. But how robust is this interaction specificity to mutation? Can individual mutations lead to cross-talk or do paralogs diverge enough to provide a mutational “buffer” against cross-talk? To address these questions in the context of a family of bacterial signaling proteins, we built mutant libraries that produce all single substitutions of the kinase EnvZ and then screened for cross-talk to noncognate proteins. Strikingly, we find that many substitutions can produce cross-talk, meaning that these pathways typically exhibit only “marginal specificity” and demonstrate that this restricts their evolvability.

The process of gene duplication and divergence fuels the evolution of proteins with new functions (1). This fundamental mechanism has created large paralogous protein families within all clades of life (2, 3). However, the expansion of these protein families presents a challenge when members are required to bind distinct interaction partners (4–8). Given their highly similar structures and sequences, how do the individual members of such families maintain different interaction specificities? And, do paralogs constrain each other’s evolution?

Answers to these questions lie in the nature of the sequence space relevant to such paralogous families. This sequence space is defined by the set of residues governing the interaction specificity of paralogs and their binding partners. In sequence space, each paralog must reside within a specific “niche,” defined here as the set of sequences capable of interacting with its binding partner(s). A given paralog may also have to avoid the niches of other proteins within this space to maintain interaction specificity. How much constraint is posed by other paralogs depends on the size, distribution, and extent of overlap of niches within sequence space.

**Fig. 1.**
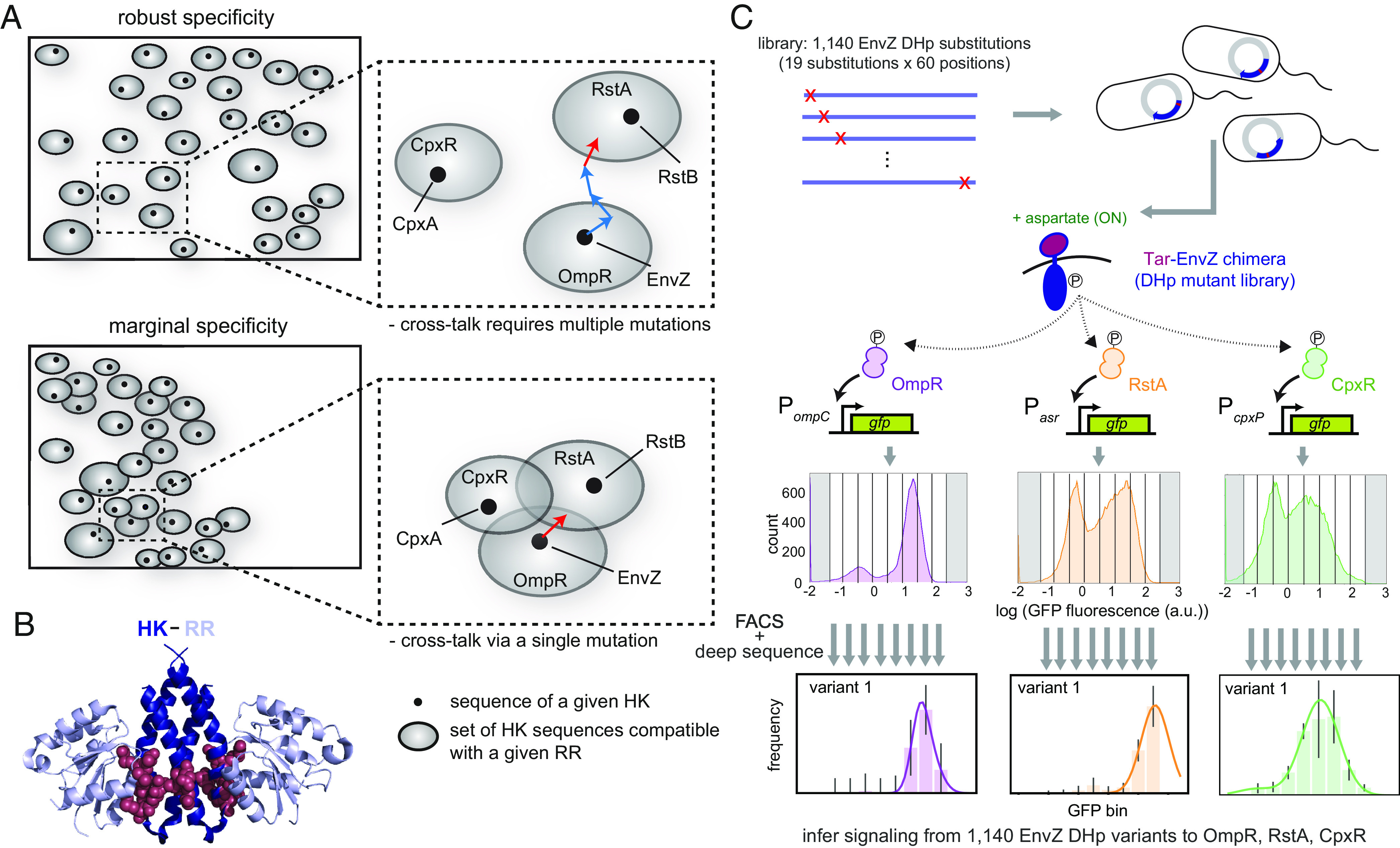
Assessing the density of local sequence space. (*A*) Sequence space diagram in which rectangles represent the space of all possible HK sequences, dots represent extant paralogs in a species (HKs in *E. coli*), and gray spheres represent the set of HK sequences that interact with a given RR. HK paralogs must interact with their cognate RR, i.e., be within the niche of that RR, but avoid the niches of noncognate RRs. *Top*: robust specificity model, where niches are generally well separated in sequence space. *Bottom*: marginal specificity model, where niches are separated just enough to avoid cross-talk, but often overlap such that local sequence space can be crowded. *Insets* show the arrangement of niches for OmpR, RstA, and CpxR. In the robust specificity model, multiple mutations (depicted as arrows) to an HK such as EnvZ are required to introduce cross-talk; in the marginal specificity model, a single mutation may introduce cross-talk. (*B*) Model of an HK–RR complex. RR chains (light blue) from PDB ID 3DGE are positioned relative to the structure of EnvZ (5B1N, deep blue) using the DHp domains in each structure for alignment. HK positions that coevolve with positions in the RR are shown as red spheres. (*C*) A library of single-substitution EnvZ variants was transformed into three GFP reporter strains that read out activation of OmpR (P*_ompC_*), RstA (P*_asr_*), or CpxR (P*_cpxP_*). The resulting populations showed a distribution of GFP fluorescence and were sorted into eight bins based on GFP level. Populations from each bin were deep sequenced, the frequency of variants in each bin was calculated, and profiles were fit to Gaussians to extract the peak fluorescence of each variant. Error bars in frequency profiles indicate SD from three replicates.

Prior work demonstrated that the sequence space of some paralogous protein families is sparsely occupied, with ample room for new members, based on the observation that new, synthetic proteins could be readily discovered or introduced without cross-talk to existing systems (9–12). However, the overall distribution of niches for extant paralogs in sequence space is not known, and there are two general possibilities. First, niches could be widely distributed throughout sequence space. Due to either selection pressure (13–18) or the drift of sequences over evolutionary time, individual niches may have moved far apart in space. This would result in “robust specificity” in the sense that cross-talk between paralogs would require multiple substitutions (Fig. 1 *A*, *Top*). Alternatively, niches for extant paralogs could be clustered and partially overlapping (Fig. 1 *A*, *Bottom*), creating crowded local regions of sequence space despite overall sparsity. This could result in “marginal specificity” such that individual substitutions could, in principle, produce cross-talk. Such marginal specificity is akin to the well-documented marginal stability of proteins in which proteins are often just above a threshold stability level needed for folding (19–23). This marginal stability arises because evolution does not select for additional stability once a protein can stably fold. A consequence, or reflection, of marginal stability is that individual substitutions can lead to unfolding. Marginal specificity could similarly arise if recently duplicated paralogs are only under selective pressure to separate in sequence space just enough to prevent unwanted cross-talk, with no pressure to further diverge and enhance the robustness of specificity.

To distinguish between these models of specificity, we investigated two-component signaling pathways, the most prevalent form of signal transduction system in bacteria, with most species encoding dozens of paralogous pathways (24). The typical pathway consists of a histidine kinase (HK) that detects a signal, autophosphorylates, and then transfers a phosphoryl group to a cooperonic, cognate response regulator (RR). The phosphorylated RR elicits a cellular output, often by regulating gene expression (25). The HK also dephosphorylates its cognate RR in the absence of signal (26). There is typically very high structural and sequence similarity between paralogs in the domains responsible for HK–RR interactions: the dimerization and histidine phosphotransfer (DHp) domain of the HK and the receiver domain of the RR (27). However, interactions between cognate HK–RR pairs are highly specific in vivo and in vitro, with little cross-talk between noncognate partners (28). HK–RR specificity is determined primarily by a subset of residues that strongly coevolve (Fig. 1*B* and *SI Appendix*, Fig. S1*A*) (29, 30). These residues are found at the HK–RR interface and, when collectively swapped from one system to another, are often sufficient to rewire interaction specificity (20, 29). Introducing multiple substitutions at these key residues can produce cross-talk between noncognate proteins that is severely detrimental to cellular fitness in certain conditions (4). However, how likely individual substitutions are to produce cross-talk has not been systematically probed. Thus, whether two-component signaling pathways exhibit marginal or robust specificity is not yet known.

## Results

### A High-Throughput Method for Assessing Cross-Talk between Signaling Pathways.

To assess paralog specificity and determine how crowded sequence space is locally, we focused on the *Escherichia coli* two-component signaling systems EnvZ–OmpR, RstB–RstA, and CpxA–CpxR (Fig. 1*A*). These three systems are widespread in β- and γ-proteobacteria, likely resulting from two ancient duplication and divergence events that occurred ~2 billion years ago in their common ancestor (31) (*SI Appendix*, Fig. S1 *B* and *C*). To examine the occupancy of sequence space immediately surrounding EnvZ, we sought to measure the ability of variants harboring each possible single substitution in the DHp domain to activate the cognate regulator OmpR and the noncognate regulators RstA and CpxR. To monitor activation, we generated strains in which a green fluorescent protein (GFP) reporter is expressed from a known OmpR-, RstA-, or CpxR-regulated promoter (32–34) (Fig. 1*C* and *SI Appendix*, Fig. S2*A*). Because the native signal for EnvZ is not known, we deleted *envZ* in each strain and introduced *taz*, which encodes a chimeric receptor containing the aspartate-sensing domain of the chemoreceptor Tar fused to the cytoplasmic, signaling domains of EnvZ (33, 35, 36) (Fig. 1*C*). Taz drives robust (~14-fold), signal-dependent induction of our OmpR reporter, but not the RstA or CpxR reporters, reflecting the limited cross-talk between the three wild-type pathways (*SI Appendix*, Fig. S2 *B* and *C*). For simplicity, we refer to the wild-type Taz construct as EnvZ. To assess cross-phosphorylation of the noncognate regulators RstA and CpxR, we deleted *rstB* and *cpxA* from each strain to prevent the phosphatase activity of these kinases from counteracting any phosphorylation of RstA or CpxR by EnvZ (33) (*SI Appendix*, Fig. S2*A*).

**Fig. 2.**
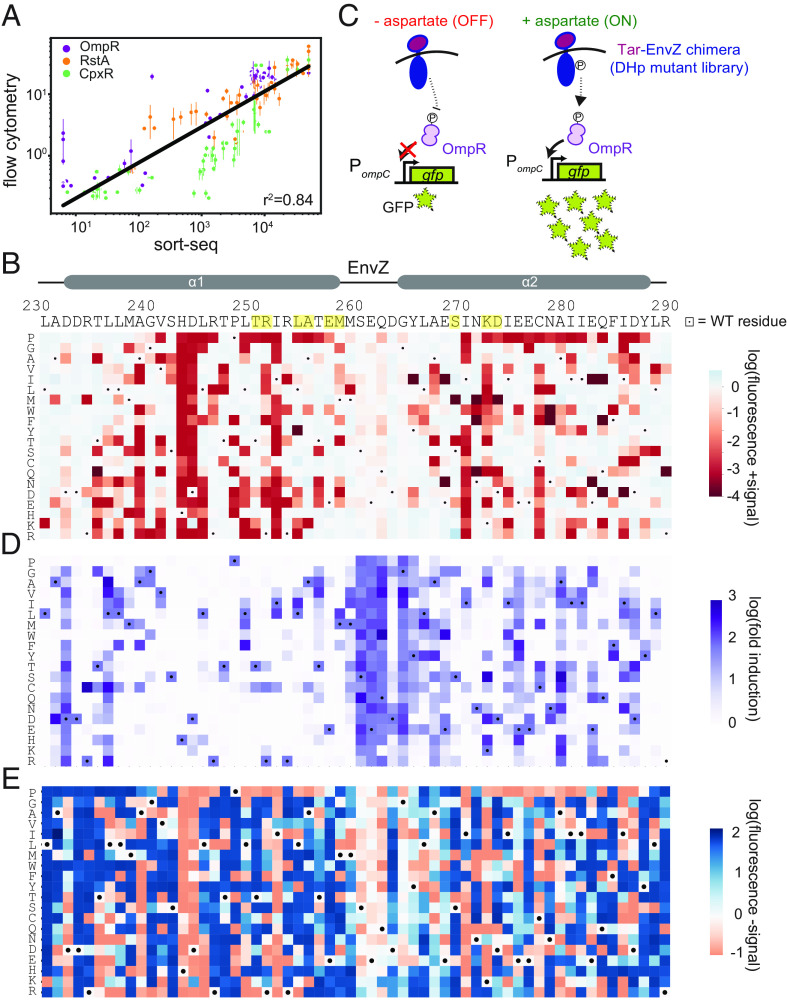
Sort-seq reveals the landscape of mutational tolerance in EnvZ-OmpR signaling. (*A*) Correlations between fluorescence values obtained by Sort-seq and by individual-clone flow cytometry. Error bars indicate SD from two independent biological replicates. Individual Pearson’s coefficients for the three reporters were r^2^ = 0.91 (OmpR), 0.92 (RstA), and 0.83 (CpxR). (*B*) Heatmap of OmpR reporter data with columns representing positions along the EnvZ DHp sequence; yellow highlights indicate coevolving residues. Rows indicate specific amino acids introduced at each position. Dots mark wild-type residues. Color-coded values represent log_10_(fluorescence) of each variant in the +signal condition. Wild-type EnvZ is set to white (blue represents increases in fluorescence, red shows decreases). (*C*) Diagram illustrating induction for the OmpR reporter. In low aspartate conditions, wild-type EnvZ is a phosphatase, removing phosphoryl groups from OmpR and leading to low GFP levels. In high aspartate conditions, wild-type EnvZ is a kinase, phosphorylating OmpR and driving high GFP production. (*D*) Same as (*B*) but with purple color indicating the log_10_(fold induction) value for each variant at each position. (*E*) Same as (*B*) but for the −signal condition. Wild-type EnvZ is set to white (blue represents increases in fluorescence, red represents decreases). Many variants have increased fluorescence in this condition suggesting that they have reduced phosphatase activity and are constitutively active.

To focus our investigation on the local sequence space immediately surrounding EnvZ, we performed deep mutational scanning (37, 38). We constructed a library of all 1,140 single substitutions in the 60 amino acid DHp domain of EnvZ and then used a high-throughput screening approach, Sort-seq (9), to measure interaction with OmpR, RstA, and CpxR (Fig. 1*C*). We transformed the library into each reporter strain and then grew cells in the presence or absence of signal (aspartate) for 3 h before sorting cells into bins based on their fluorescence. The plasmids encoding *envZ* in cells from each bin in each condition were deep sequenced to determine the frequency of each variant, with frequency profiles fit to a Gaussian to extract the peak fluorescence values (9) (Fig. 1*C* and *SI Appendix*, Fig. S2 *D–J*). To validate these values, 30 variants spanning the range of output fluorescence values for each regulator in both conditions were measured individually using flow cytometry, and the median fluorescence was compared to the values obtained from Sort-seq. For each reporter, there was a high correlation (R^2^ > 0.8) between the values obtained from Sort-seq and flow cytometry (Fig. 2*A*). We also purified a selection of 14 EnvZ variants and used ^32^P-based phosphotransfer assays to demonstrate that the activities toward the regulators seen in vivo were recapitulated in vitro (*SI Appendix*, Fig. S3).

### Deep Mutational Scanning Reveals the Marginal Specificity of Paralogs.

To visualize our deep mutational scanning data for transfer to the cognate regulator OmpR, we generated a heatmap displaying the fluorescence levels in the presence of inducer (+signal) for each possible substitution at each position in the DHp domain of EnvZ (Fig. 2*B*). Most (81%) substitutions retained levels similar (within 5-fold) to wild-type EnvZ, indicating that they retain kinase activity (Fig. 2*B*). When visualizing fold-induction value (fluorescence +/− signal, Fig. 2*C*), 76% of substitutions eliminated or reduced the fold-induction relative to the wild-type (Fig. 2*D*). A clear exception was within the loop region connecting the α1 and α2 helices of the DHp where a wide range of substitutions was tolerated. The loss of signal responsiveness for many variants may result from reduced phosphatase activity in the absence of a signal, producing a constitutively active kinase (Fig. 2*E*).

**Fig. 3.**
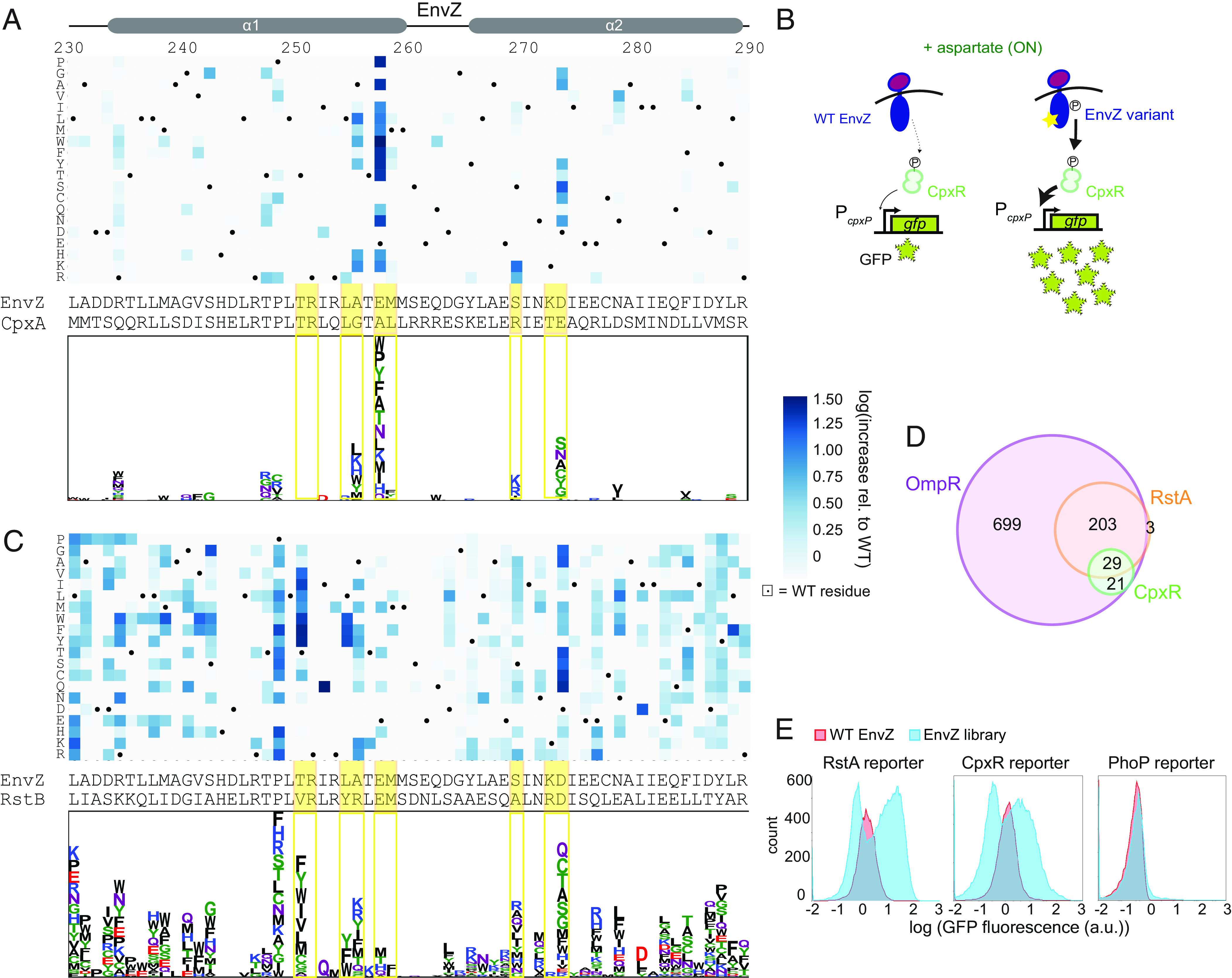
EnvZ exhibits marginal specificity, reflecting a crowded local sequence space. (*A*) *Top*: Heatmap of CpxR reporter data where values represent increases in GFP fluorescence relative to wild-type EnvZ in the +signal condition. Dots mark wild-type residues. EnvZ and CpxA DHp sequences are shown below. Yellow highlighted positions mark coevolving residues. Values were normalized by increases in fluorescence toward OmpR, which may reflect nonspecific effects of a substitution on expression level or kinase activity that increase activity toward all RRs. *Bottom*: Logo transformation of heatmap where the height of a letter represents the increase in fluorescence relative to wild-type for that amino acid substitution at that position. Letters are stacked in ranked order. (*B*) Diagram illustrating fluorescence measurements for CpxR reporter. Wild-type EnvZ shows low levels of kinase activity, leading to low GFP levels. EnvZ variants (as indicated by the star) may show increased kinase activity, driving high GFP production. (*C*) Same as (*A*) except for RstA reporter data. (*D*) Overlap of single-substitution EnvZ variants with activity toward different RRs. The OmpR set contains variants with kinase activity for OmpR comparable to wild-type EnvZ (within 5-fold). The RstA and CpxR sets contain variants with ≥5-fold increases in activity toward RstA or CpxR relative to wild-type EnvZ. (*E*) Histograms of GFP fluorescence distributions for the RstA, CpxR, and PhoP reporters. Red populations are for wild-type EnvZ, blue populations are transformations of the single mutant library. Blue library populations for RstA and CpxR reporters are replicated from Fig. 1*C* for comparison to PhoP.

To quantify cross-talk from each EnvZ variant to CpxR, we generated a heatmap showing the increase in fluorescence of the CpxR reporter in the +signal condition relative to that of wild-type EnvZ, which was set to 0 (Fig. 3*A*). Increases in fluorescence represent increased kinase activity, which could disrupt signaling fidelity and constitute detrimental cross-talk (Fig. 3*A* and *SI Appendix*, Fig. S4 *A–C*). No single substitution produced an EnvZ variant with signal-responsive activity toward CpxR, possibly due to reduced in vivo phosphatase activity. Although the majority of substitutions in EnvZ did not increase cross-talk to CpxR, a small number of substitutions showed fluorescence values increased as much as 30-fold relative to wild-type EnvZ. This level of activation was similar to that of a chimera of the Tar sensor domain fused to the cytoplasmic signaling domains of CpxA, the cognate HK of CpxR (*SI Appendix*, Fig. S4*D*). The cross-talk-inducing substitutions occurred primarily at the coevolving positions previously shown to be important for HK–RR interaction specificity (29, 30). For instance, at Ala255, Glu257, and Asp273, multiple substitutions with dissimilar biochemical characteristics caused cross-talk, suggesting that the native residues at these positions serve as negative design elements that prevent cross-talk to CpxR. At Ser269, only two substitutions, the positively charged residues Arg and Lys, caused substantial increases in cross-talk, suggesting that these residues may promote an interaction with CpxR that the wild-type Ser residue cannot. Notably, the corresponding residue of CpxA is Arg, consistent with positive charge at this position facilitating interaction with CpxR. At other coevolving positions, including Thr250, Arg251, Leu254, and Met258, no substitutions substantially increased cross-talk. These residues are each identical or biochemically similar in EnvZ and CpxA, consistent with them not being involved in insulating these two pathways.

We also assessed cross-talk to RstA (Fig. 3*C*). The strongest cross-talk-inducing substitutions again tended to occur at the coevolving residues, with similar patterns seen as with CpxR. At some coevolving positions in which EnvZ differs significantly from the corresponding residue of RstB, such as Thr250, Ala255, and Ser269, multiple biochemically distinct substitutions led to substantial cross-talk, indicating that these residues act as negative design elements with respect to RstA. At Leu254, only aromatic residues caused cross-talk suggesting that they form specific contacts with RstA that enhance its interaction with EnvZ; notably, RstB features a Tyr at this position. In a strikingly different pattern than we observed for CpxR, there were also a large number of substitutions at noncoevolving positions that caused cross-talk, which are discussed below.

In total, there were 21, 206, and 29 substitutions that increased cross-talk more than 5-fold toward CpxR, RstA, or both, respectively (Fig. 3*D*). Similar patterns and relative numbers of variants were seen at thresholds of 3- and 10-fold, indicating that our results are robust to the precise threshold used (*SI Appendix*, Fig. S4 *E*–*G*). The observation that many individual substitutions can readily produce cross-talk indicates that EnvZ exhibits marginal, rather than robust, specificity with respect to CpxR and RstA. Considering both CpxR and RstA, we found that EnvZ variants containing the corresponding residue of the respective cognate kinase (CpxA and RstB, respectively) were more likely to exhibit cross-talk relative to other substitutions (*P* = 0.004, odds ratio = 2.62, Fisher’s exact test; *SI Appendix*, Fig. S5*A*). However, there were still a large number of EnvZ substitutions that did not resemble the corresponding residue on the other kinases but still caused cross-talk. Together, these findings demonstrate that mutating the EnvZ sequence to mimic RstB or CpxA is not the only way to generate cross-talk to RstA or CpxR (*SI Appendix*, Fig. S5*B*).

We also found many substitutions that decreased the activity of EnvZ toward either or both noncognate regulators, without decreasing activity toward the cognate regulator OmpR (*SI Appendix*, Fig. S6 *A–C*). We likely observed this only because EnvZ is overexpressed in our assay; at native levels, EnvZ shows no detectable activity toward RstA and CpxR (*SI Appendix*, Fig. S6 *D* and *E*). However, this finding does suggest that cross-talk between these pathways has not been eliminated and instead has only been reduced to such a level that it has no effect on fitness. The notion that interactions with noncognate proteins could be reduced further by many different single substitutions emphasizes that only marginal specificity has been selected for between these systems.

We hypothesized that the marginal specificity of EnvZ–OmpR, RstBA, and CpxAR reflects their phylogenetic history as closely related paralogs. Duplication events that led to the emergence of these three systems were likely followed by changes in specificity sufficient to insulate these pathways, such that they could carry out distinct functions, but leaving them close in sequence space. In contrast, less closely related pathways are likely further apart in sequence space such that specificity is more robust. To test this idea, we transformed the library of EnvZ variants into a reporter strain for the more distantly related regulator PhoP, for which the cognate kinase is PhoQ (*SI Appendix*, Fig. S1*C*), and performed flow cytometry. Unlike with the RstA and CpxR reporter strains, there was no subpopulation of cells showing significantly increased fluorescence relative to wild-type EnvZ, indicating that no or very few single substitutions in EnvZ cause substantial cross-talk to the distantly related PhoP (Fig. 3*E*).

### The Extent of Marginal Specificity Reflects the Evolutionary History of Paralogs.

**Fig. 4.**
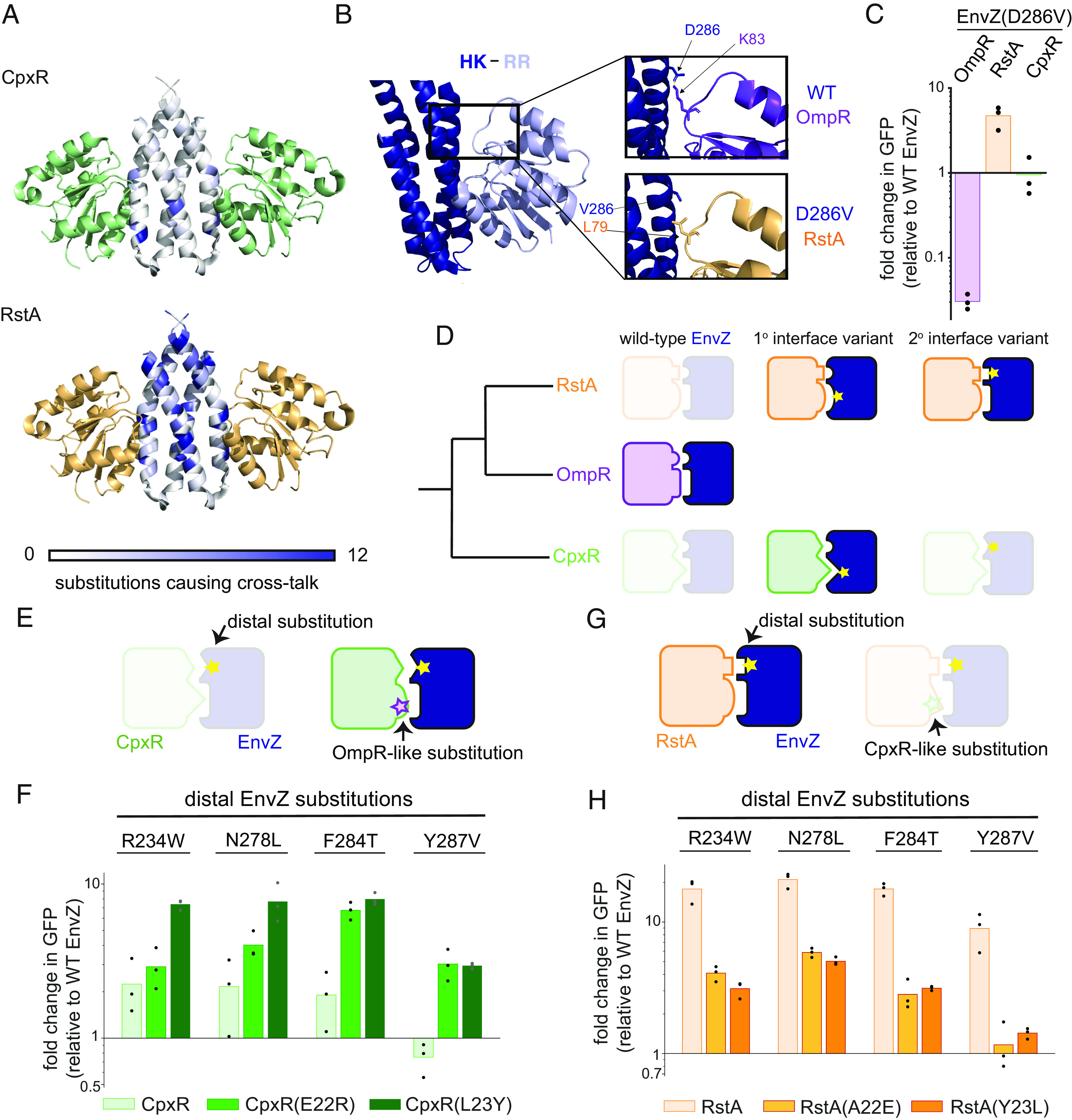
The degree of marginal specificity reflects phylogenetic relatedness between paralogs. (*A*) Number of substitutions causing ≥5-fold cross-talk to either CpxR (green) or RstA (orange) at each position in EnvZ is color-coded and mapped onto the model complex structure in the phosphatase state. (*B*) Active phosphotransfer state structure with HK in deep blue and RR in light blue (PDB: 5IUL). *Insets* show wild-type EnvZ and OmpR, or EnvZ(D286V) and RstA residues modeled onto this structure. Side chains were placed in the most preferred rotamers for interaction. (*C*) Fold change in fluorescence +signal for EnvZ(D286V) relative to wild-type EnvZ for each reporter strain. n = 3 biological replicates. (*D*) Model in which faded protein pairs represent unsuccessful interaction and full-color pairs represent successful interaction. RstA and CpxR have diverged sufficiently from OmpR to prevent cross-talk with wild-type EnvZ. However, RstA retains enough compatibility that substitutions at either the primary or secondary interface (yellow stars) can produce cross-talk. By contrast, CpxR has diverged such that its primary interface is fundamentally incompatible with EnvZ, and only substitutions that suppress incompatibility at this interface create cross-talk. (*E*) Model in which distal substitutions do not create cross-talk to wild-type CpxR but substitutions at the CpxR primary interface that increase its compatibility with EnvZ can increase sensitivity to distal substitutions. (*F*) Fold changes in +signal fluorescence relative to wild-type EnvZ for four distal single substitutions in EnvZ, against wild-type CpxR and CpxR variants with OmpR-like primary interface substitutions: E22R and L23Y. n = 3 biological replicates. (*G*) Model in which distal substitutions in EnvZ can produce cross-talk to RstA, but substitutions at the RstA primary interface that reduce its compatibility with EnvZ decrease its sensitivity to these distal substitutions. (*H*) Fold changes in +signal fluorescence relative to wild-type EnvZ for four distal single substitutions in EnvZ, against wild-type RstA and RstA variants with CpxR-like primary interface substitutions: A22E and Y23L. n = 3 biological replicates.

Collectively, our results suggest that the occupancy of sequence space reflects the evolutionary history of paralogs and that cross-talk is most likely to occur between more closely related systems. Because EnvZ–OmpR is more closely related to RstBA than to CpxAR (*SI Appendix*, Fig. S1*C*), this model may explain why many more single substitutions can cross-talk to RstA than CpxR (Fig. 3*C*). To assess the spatial distribution of substitutions that induced cross-talk, we mapped these substitutions onto a modeled structure of the EnvZ–OmpR complex in the phosphatase state, i.e., the state in which an HK is thought to promote dephosphorylation of its phosphorylated cognate RR (Fig. 4*A*). This is the state most commonly seen in two-component complex structures (39–41), likely due to its rigidity facilitating crystallization. Substitutions that increased cross-talk to CpxR in our assay were generally on the surface of the HK dimer at the interface with the RR, which we refer to here as the primary interface. As noted above, these cross-talking substitutions largely involved residues known to coevolve in HK–RR pairs (29, 30) (Fig. 3*A*). In contrast, the substitutions that caused cross-talk to RstA mapped all over the DHp domain, including at positions distal to the interface and even some within the core of the dimer (Fig. 4*A*).

We also modeled EnvZ–OmpR in the suspected kinase, or phosphotransfer, state, using the single complex that has been solved (42). In this structure, the N- and C-terminal portions of the DHp domain, which form the upper part of the dimeric four-helix bundle, reside closer to the β4–α4 loop of the RR (Fig. 4*B*). Interactions at this secondary interface may explain the cross-talk behavior of some substitutions distal to the primary interface of both states. For example, the substitution D286V in EnvZ caused a ~50-fold decrease in activity toward OmpR but a ~5-fold increase in activity toward RstA (Fig. 4*C*). Examining EnvZ–OmpR modeled onto the kinase-state structure, Asp286 is positioned such that it can form a salt bridge with Lys83 on the β4–α4 loop of OmpR (Fig. 4*B*). Substituting this Asp with a Val eliminates this favorable interaction, which likely explains the decreased transfer from EnvZ(D286V) to OmpR. The residue corresponding to Lys83 in RstA is Leu (L79), possibly explaining why a hydrophobic Val in place of Asp286 in EnvZ is more favorable for this interaction than the charged Asp (Fig. 4*B*).

Although additional interactions at the secondary interface may explain some of our results, they are unlikely to explain the effects of cross-talk–inducing substitutions within the DHp core. Such substitutions presumably impact the primary or secondary interface allosterically to enhance interaction with the noncognate RstA. Importantly, almost no substitutions in the DHp core or at positions away from the primary interface created cross-talk with the less closely related CpxR (Fig. 4*A*). Thus, we hypothesized that the primary interfaces of EnvZ–OmpR and RstB–RstA have diverged enough to reduce cross-talk between wild-type EnvZ and RstA but are still sufficiently compatible that a single substitution at a distal site can result in cross-talk. By contrast, the primary interface of CpxA–CpxR is far enough diverged from EnvZ–OmpR that only substitutions at this interface can yield large enough effects to produce cross-talk (Fig. 4*D*).

This hypothesis predicts that increasing the compatibility of the primary interface between EnvZ and CpxR should increase the propensity of distal substitutions in EnvZ to cause cross-talk (Fig. 4*E*). To test this idea, we sought to substitute interfacial residues in CpxR with those found in OmpR and measure whether there is epistasis between these substitutions and distal substitutions in EnvZ. We focused on two coevolving interface positions at the primary interface of CpxR that differ significantly from the corresponding residues in both OmpR and RstA: Glu22 and Leu23 (*SI Appendix*, Fig. S7*A*). We substituted each of these residues individually with the corresponding residue found in OmpR, making CpxR variants E22R and L23Y, and then tested these variants for interaction with wild-type EnvZ and a selection of EnvZ variants with substitutions (R234W, N278L, F284T, and Y287V) that are distal to the primary interface and caused cross-talk to RstA but not CpxR (*SI Appendix*, Fig. S7 *B* and *C*). For each EnvZ variant, there was significantly more cross-talk to the CpxR primary interface variants than to wild-type CpxR (Fig. 4*F* and *SI Appendix*, Fig. S7*D*). This positive epistasis between interfacial residues of CpxR and interface-distal residues of EnvZ suggests that improved compatibility between EnvZ and CpxR at the primary interface increases the susceptibility of CpxR to cross-talk induced by single substitutions elsewhere in EnvZ.

Our model also predicts that decreasing the compatibility of the primary interface between EnvZ and RstA could have the opposite effect, decreasing the propensity of interface-distal substitutions in EnvZ to cause cross-talk (Fig. 4*G*). To test this prediction, we substituted the same primary interface positions in RstA to match those of CpxR, A22E, and Y23L, and then tested these RstA variants for interaction with wild-type EnvZ and the same EnvZ variants as above. In each case, cross-talk to RstA caused by distal substitutions in EnvZ was significantly reduced for both RstA A22E and Y23L (Fig. 4*H* and *SI Appendix*, Fig. S7 *E* and *F*). This negative epistasis between interfacial residues of RstA and interface-distal residues of EnvZ further supports our model that the propensity for cross-talk between noncognate proteins depends on latent compatibility between the proteins at the primary interface, which reflects the evolutionary history of the paralogs.

### Avoiding Cross-Talk Is a Pervasive Selective Pressure Shaping Sequence Space Occupancy.

We conclude that EnvZ exhibits marginal specificity, reflecting a crowded local region of sequence space. This further suggests that the specificity of extant two-component signaling paralogs can be fragile, easily disrupted by single substitutions throughout the kinase. We sought to assess whether this marginal specificity has broadly affected EnvZ evolution.

**Fig. 5.**
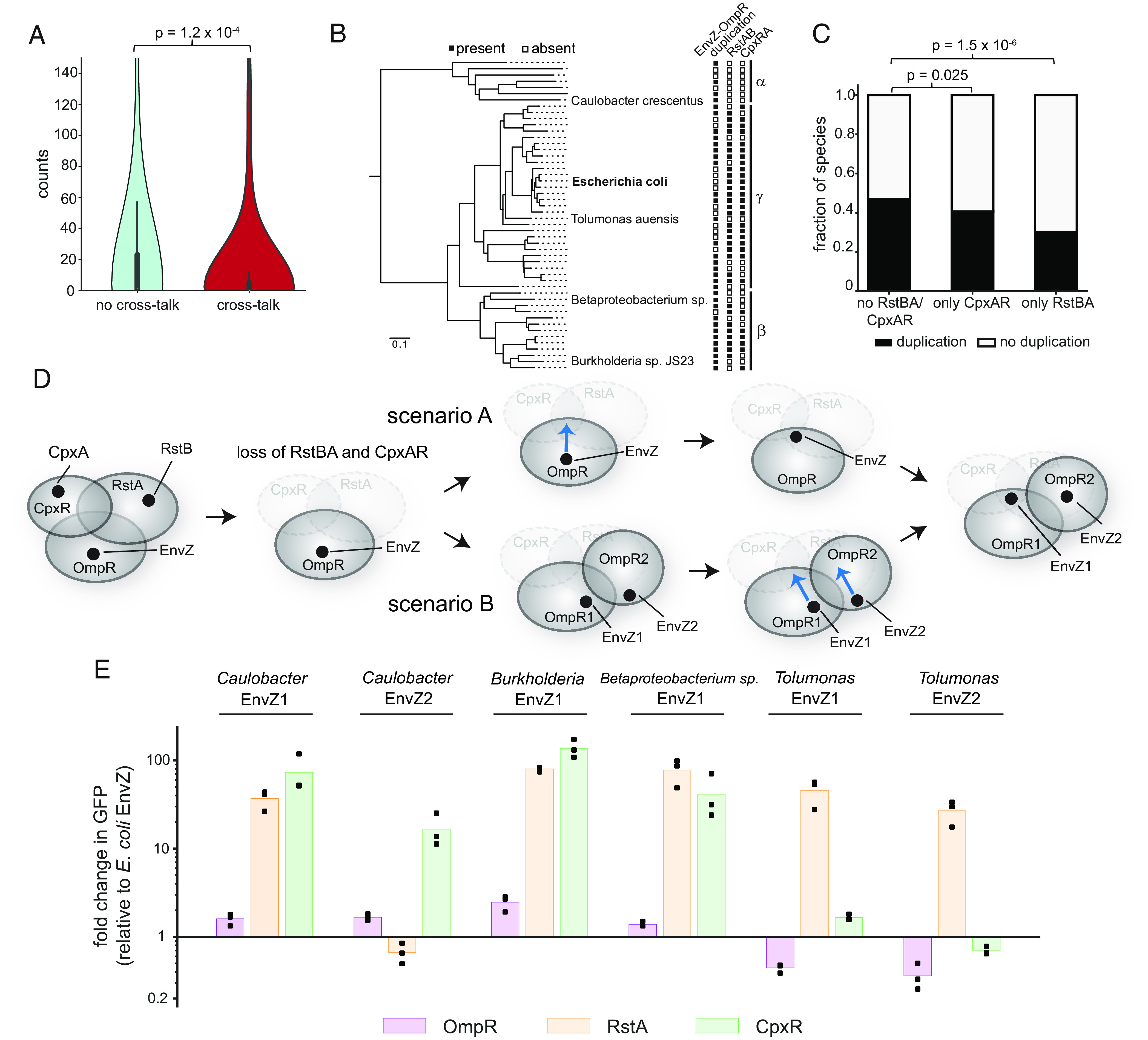
A densely occupied local sequence space constrains the evolution of two-component systems. (*A*) Violin plots show the distributions of counts of single substitutions found at the equivalent position in 1,019 EnvZ orthologs from species that also have RstBA and CpxAR. Counts are shown for two categories of substitution: those which do produce cross-talk to either RstA or CpxR and those which do not (see Fig. 3, *P* = 1.2 × 10^−4^, Kolmogorov–Smirnov test). The inner box shows the quartiles and the whiskers show the range except for outliers. (*B*) Tree of a subset of species indicating whether they have RstBA and CpxAR and whether they have EnvZ–OmpR duplications. Closed and open squares indicate presence and absence, respectively. (*C*) Fraction of species that either do not have RstBA or CpxAR, have only CpxAR, or have only RstBA, that have duplications of EnvZ–OmpR (*P* = 0.025 for CpxAR loss, *P* = 1.5 × 10^−6^ for RstBA loss, Fisher’s exact test). (*D*) Sequence space diagram illustrating how species that have lost RstBA and CpxAR may relax the selection pressure against entering regions of sequence space that would previously have resulted in cross-talk, also freeing up sequence space for EnvZ–OmpR duplications. Blue arrow indicates a single mutation in EnvZ that would be tolerated only in species that have lost CpxR and RstA. This could occur before duplication (scenario A) or after (scenario B). (*E*) Fold changes in fluorescence relative to *E. coli* EnvZ for six EnvZ homologs from four distantly related species, for *E. coli* OmpR, RstA, and CpxR reporters. n = 3 biological replicates.

First, we used HMMER to identify and align a set of 5,751 EnvZ orthologs from a wide range of proteobacteria. We then calculated the frequencies at which residues that caused cross-talk from *E. coli* EnvZ to either CpxR or RstA appear at the equivalent position in EnvZ orthologs from other species that also have RstBA and CpxAR. These cross-talk-inducing residues were found less frequently than residues that do not cause cross-talk (*P* = 1.2 × 10^−4^, D = 0.15, Kolmogorov–Smirnov test, Fig. 5*A*). We also found that a higher proportion of cross-talk–inducing residues were completely absent at the equivalent positions in EnvZ orthologs (*P* = 6.2 × 10^−4^, odds ratio = 0.15, Fisher’s exact test, *SI Appendix*, Fig. S8*A*). These observations suggest that even averaged across a large number of sequence backgrounds, the substitutions that we found to cause cross-talk may have been selected against, leading to their lower prevalence among EnvZ orthologs.

To test whether the differences seen were specific to EnvZ, we aligned ortholog sequences of three other HKs, PhoR, YehU, and BarA, which are increasingly distantly related to EnvZ (*SI Appendix*, Fig. S1*B*). For YehU and BarA, there was no significant difference between the frequencies of the two classes of residues at the equivalent positions (*P* = 0.80, D = 0.043 for YehU, *P* = 0.18, D = 0.074 for BarA, Kolmogorov–Smirnov test, *SI Appendix*, Fig. S8 *B* and *C*) or the proportion of residues that were absent (*P* = 0.68, odds ratio = 0.94 for YehU, *P* = 0.17, odds ratio = 0.82 for BarA, Fisher’s exact test, *SI Appendix*, Fig. S8 *D* and *E*). For PhoR, there was a significant difference between the two categories, although it was smaller than the difference observed for EnvZ (*P* = 0.0016, D = 0.13, Kolmogorov–Smirnov test, *P* = 0.003, odds ratio = 0.12, Fisher’s exact test, *SI Appendix*, Fig. S8 *F* and *G*). These results suggest that more closely related kinases share some of the same sequence features and selective pressures faced by EnvZ, but these pressures do not apply to more distantly related kinases.

Although many γ-proteobacteria, like *E. coli*, have EnvZ–OmpR, RstBA, and CpxAR, several species have lost one or more of these systems (Fig. 5*B*). We wondered if losing RstBA or CpxAR relaxes the selection pressure against cross-talking mutations and allows drift of EnvZ into the regions of sequence space they previously occupied. Indeed, we found that cross-talk–inducing substitutions are seen at higher frequencies in EnvZ orthologs from species that have lost RstBA or CpxAR (*P* = 0.048, D = 0.11, Kolmogorov–Smirnov test, *SI Appendix*, Fig. S8*H*). Additionally, we found that species that have lost RstBA and CpxAR were more likely to have duplicated EnvZ–OmpR (*P* = 0.025, odds ratio = 0.77 for CpxAR loss, *P* = 1.5 × 10^−6^, odds ratio = 0.49 for RstBA loss, Fisher’s exact test, Fig. 5*C*). This finding suggests that the presence of these systems, particularly the most closely related RstBA, limits the sequence space available to EnvZ and thus constrains the ability of EnvZ–OmpR to duplicate and establish a new system (Fig. 5*D*).

We further predicted that in species lacking RstBA and CpxAR in which EnvZ–OmpR had duplicated, the EnvZ paralogs may now occupy sequence space made available by the loss of the other systems (Fig. 5*D*). To test this prediction, we chose four species, distantly related to each other and to *E. coli*, in which RstBA and CpxAR were independently lost and EnvZ had been duplicated (Fig. 5*B*). Each of the resulting EnvZ homologs had residues that caused cross-talk in the context of *E. coli* EnvZ (*SI Appendix*, Fig. S8*I*). These residues occurred at both primary and secondary interface positions, as well as in core residues of the DHp domain. We cloned and expressed each homolog in our reporter strains for *E. coli* OmpR, RstA, and CpxR and then measured GFP expression relative to that seen with *E. coli* EnvZ. For each species considered, one or both EnvZ homologs showed high levels of cross-talk to *E. coli* RstA, CpxR, or both (Fig. 5*E*). This result strongly supports the notion that without RstBA and CpxAR, EnvZ duplicates commonly enter the sequence space freed up by the loss of these other systems. This finding further demonstrates how the presence of closely related paralogs, and the consequent marginal specificity, has constrained EnvZ evolution.

## Discussion

Our findings demonstrate that the distribution of niches in sequence space of paralogous two-component signaling systems is not globally optimized for specificity or selected for robustness to mutation. Although the requirement for only a marginal level of specificity during the early establishment of duplicates may facilitate their evolution, it comes at the cost of future constraint on the emergence of additional duplications. Over time, due to drift and movement catalyzed by subsequent duplications, paralogous systems can continue to move apart in sequence space such that more distantly related systems are robustly insulated. However, systems like EnvZ–OmpR, CpxA–CpxR, and RstB–RstA, with ~2 billion years of divergence continue to exhibit marginal specificity. Thus, this drift is likely slower than the rate at which additional duplication events occur, such that the marginal specificity of existing paralogs will constrain the evolution of new duplicates when they emerge.

Examples of single substitutions in proteins creating nonspecific interactions have also been found in other unrelated paralogous families, in both bacteria and eukaryotes (5, 7, 43). These anecdotal examples suggest that the principle of marginal specificity may generally apply during evolution. In many families, paralogs may share functional binding partners and not require all interactions to be fully specific. However, the marginal specificity principle could apply in any case where there is a cost incurred by a nonspecific interaction. Such cases are likely to occur between most paralogs whose functions are nonredundant and involve protein–protein interactions.

Our results demonstrate how the nature of evolution, in only selecting for “good enough,” rather than fully optimized systems, can result in small margins in specificity and constrain the subsequent evolvability of a paralogous family. This principle also applies to protein stability (21, 44), abundance (45), and localization and assembly properties (46). In each case, a large proportion of substitutions can disrupt the relevant property, suggesting that robustness has not evolved in these traits. The fragility of these properties has important implications for disease pathogenesis. For example, single substitutions that disrupt the stability and abundance of tumor suppressor proteins are implicated in cancer (45), and single substitutions that affect assembly properties of proteins can drive hemoglobinopathies such as sickle cell anemia. The same appears to be true of specificity, where single “network-attacking” substitutions that alter the specificity profile of human kinases are thought to disrupt cellular signaling networks and contribute to cancer progression (47).

In addition to shedding light on the fundamental mechanisms of evolution and their consequences for paralogous proteins, our findings also have implications for protein design and directed evolution methods. Attempts to build new signaling systems while avoiding detrimental cross-talk with existing cellular systems may work better if employing randomization or mutagenesis of multiple residues, allowing “jumps” into sparsely occupied regions of sequence space, rather than methods that traverse crowded local sequence spaces by moving one mutation at a time. Overall, we demonstrate an example of marginal specificity in protein interactions that has implications for the evolvability of paralogous proteins, in both natural and synthetic settings.

## Methods

### Bacterial Strains and Media.

*E. coli* strains were grown in M9 medium (1× M9 salts, 100 μM CaCl_2_, 0.2% glucose, 2 mM MgSO_4_, with or without 5 mM aspartate). When indicated, antibiotics were used at the following concentrations: carbenicillin 50 μg/mL, kanamycin 50 μg/mL, spectinomycin 50 μg/mL, and chloramphenicol 32 μg/mL. The base strain for all studies was *E. coli* strain ML1803 (Yale BW28357 Δ*envZ*, *SI Appendix*, Table S1) (32). The OmpR reporter strain contained a p15a/cmR plasmid containing P*_ompC_*-*gfp* (32) (*SI Appendix*, Table S2 and Dataset S1). The RstA, CpxR, and PhoP reporter strains each contained additional deletions: Δ*ackA-pta* (removes a pathway that generates acetyl phosphate, which can phosphorylate RRs in the absence of a HK) and Δ*rstB,* Δ*cpxA*, or Δ*phoQ* (to prevent the cognate HKs of RstA, CpxR, or PhoP, respectively, from phosphorylating or dephosphorylating them, *SI Appendix*, Table S1), and the same reporter plasmid but with P*_asr_*-*gfp*, P*_cpxP_*-*gfp*, or P*_mgrB_*-*gfp*, respectively (*SI Appendix*, Table S2 and Datasets S2–S4).

All libraries were cloned onto a low-copy pSC101/spec^R^ plasmid in which Taz variant expression was driven by a constitutive P*_lpp_* promoter (*SI Appendix*, Table S2 and Dataset S5). Characterization of individual, specific Taz variants was done using the same plasmid. EnvZ point mutations or homolog sequences were introduced using Gibson assembly using primers DG001-066 (Dataset S6). For the experiments involving mutations in the RRs, genomic mutations were made. Deletions discussed above and genomic mutations were made using *sacB-kan^R^* cloning. The loci in the relevant reporter strains were first replaced with the *sacB-kan^R^* locus using recombination (48), and selected using kanamycin resistance. The *sacB-kan^R^* loci were then replaced using DNA fragments that corresponded to either the sequence of clean deletions, or genes with the relevant mutations, and selected using negative selection on sucrose. The relevant region of the genome was amplified by PCR and sequenced to confirm that the deletions/mutations were correct.

### Flow Cytometry Characterization.

To induce Taz, cells were grown to early exponential phase [optical density at 600 nm (OD_600_) of about 0.2] in M9 before adding aspartate to a final concentration of 5 mM. Cells were grown for 3 h and diluted 1:40 into phosphate buffered saline (PBS) with 0.5 g/L kanamycin, and fluorescence was measured on a Miltenyi MACSQuant VYB. In each cytometry experiment, three colonies of each strain were grown and induced independently and 30,000 cells were measured per replicate. FlowJo was used to analyze the data, gating on single live cells and extracting the median of the GFP distribution.

### Design and Assembly of the Taz Library.

A comprehensive single-mutant library was constructed using oligonucleotide-directed mutagenesis of the EnvZ DHp domain. To mutate each position in the Taz DHp (positions 230 to 289), two complementary 30-nucleotide primers (one sense, one antisense) were synthesized that introduce an NNS codon at the targeted position (primers DG067-186, Dataset S6). N is a mixture of A, T, C, and G, and S is a mixture of G and C. This mutagenesis strategy results in 32 possible codons, which cover all 20 amino acids. One round of PCR was carried out with one reaction containing the antisense NNS primer and DG187, a primer located 105 bp upstream of the 5′ end of the DHp domain, containing a SacI restriction site, and a second reaction containing the sense NNS primer and DG188, a primer flanking the 3′ end of the *taz* gene, containing a SalI restriction site. A second PCR round using both first-round products and both flanking primers produced the full-length double-stranded product. All reactions yielded a band of the correct size on an agarose gel, which was extracted and purified (Zymo). PCR product concentrations were quantified (NanoDrop), pooled in equimolar ratios, digested with SacI and SalI, and ligated into the pSC101/spec^R^ expression vector. Each ligation was dialyzed on Millipore VSWP 0.025-μm membrane filters for 60 min and the entire volume was electroporated into 20 μL Invitrogen One Shot TOP10 Electrocomp *E. coli* to yield ~10^6^ total transformants. Plasmids from these transformants were then purified by miniprep (Zymo), dialyzed, and electroporated into the experimental strains, yielding ~10^9^ transformants for each strain.

### Sort-seq.

For each of three replicates, 1 mL of overnight culture of the library was washed with M9 and inoculated into 50 mL of M9. Cells were grown to OD_600_ = 0.2, and each culture was split into two: Aspartate was added to a final concentration of 5 mM to one of the cultures. After 3 h, cells were diluted (1:30) into PBS containing 320 μg/mL chloramphenicol and cells were placed on ice for sorting. Cells were sorted into bins based on GFP expression on a BD FACS (flourescence activated cell sorting) Aria machine. Single live cells were isolated using the gating strategy in *SI Appendix*, Fig. S2*J*. The FITC (fluorescein isothiocyanate) voltage was adjusted so that the population spanned the range of fluorescence the machine could detect. A live histogram of FITC fluorescence was drawn and gates were spaced evenly along the log_10_(GFP) axis. For each library replicate, both the on and off cultures were sorted into eight separate bins, generating 48 total bins. Up to ~2 million cells were sorted into bins per replicate (*SI Appendix*, Fig. S2 *G*–*I*). Sorted cells were added to 2× YT medium containing chloramphenicol and spectinomycin and then grown overnight. There is a possibility of enrichment or deenrichment of variants during the overnight growth due to differences in fitness; however, we have not observed such differences during growth in rich media previously. In addition, variants would be expected to be enriched or deenriched at proportionally the same rates in each bin sorted.

### Illumina Sample Preparation.

After FACS, plasmids were purified (Zymo) from overnight cultures representing each bin from each library replicate. Two PCR reactions were performed, both using KAPA HiFi, to add Illumina sequencing adaptors and barcodes. First, DHp domain sequences were amplified for 12 cycles (95 °C for 30 s, 68 °C for 15 s, and 72 °C for 30 s) with Illumina inner amplification primers (primers DG189-198, Dataset S6). Second, purified PCR product from the first reaction was amplified in a second PCR with barcoding primers (primers DG199-224, Dataset S6) for nine cycles (95 °C for 30 s, 68 °C for 15 s, and 72 °C for 30 s). Final products were quantified (NanoDrop), normalized, combined, and sequenced on an Illumina NextSeq. For each bin, 1 to 2 million reads were collected.

### Illumina Data Processing.

Sort-seq data processing was carried out as previously described (9). The frequency of each variant in each bin was calculated by taking the fraction of reads in a given bin that corresponded to a given sequence, normalized by the fraction of cells in that given bin. For each variant, the mean frequencies in each bin across three replicates and SD were used to fit Gaussian functions to each distribution [in log_10_(GFP units)], from both the on and off sorts (SciPy optimize package). Fold-induction values were calculated as the ratio of the means between the induced and uninduced states: μ_on_/μ_off_. To assess cross-talk to CpxR and RstA, fluorescence values in the presence of inducer were normalized against increases in fluorescence toward OmpR, which may reflect nonspecific effects of a substitution on expression level or kinase activity that increase activity toward all three RRs. Gaussian fit means for each variant for each reporter can be found in Dataset S7 (OmpR), Dataset S8 (RstA), and Dataset S9 (CpxR).

### Purification of Two-Component Signaling Proteins and In Vitro Phosphotransfer Assays.

Expression and purification of EnvZ variants and RRs, and phosphotransfer experiments, were carried out as previously described (29, 30, 49). RRs were fused to a His_6_–Trx tag, and the cytoplasmic region of EnvZ (residues 222 to 451) was fused to a His_6_–MBP (maltose-binding protein) tag, expressed in BL21(DE3) cells and purified on a Ni^2+^-NTA column. For phosphotransfer reactions, the kinase was autophosphorylated for 1 h at 30 °C with [γ-^32^P]ATP (Perkin Elmer) before being combined with RRs at a 1:4 ratio (10-μL reactions contained 1 μM EnvZ and 4 μM RR). Reactions were stopped at the times noted by adding 4× Laemmli buffer with 8% 2-mercaptoethanol. The control lane had this solution added after the autophosphorylation but prior to addition of RR. HKs and RRs were separated by SDS-PAGE, gels were incubated with phosphor screens and imaged using a Typhoon imager (GE Healthcare) at 50-mm resolution. A representative image of two independent experiments is shown in *SI Appendix*, Fig. S3.

### Identification and Assembly of Ortholog Sequences and Trees.

An alignment of HKs was built by constructing a Hidden Markov Model profile from an alignment of DHp and CA domains of *E. coli* HKs (29) and searching the ProGenomes2.0 database (50) with this profile (51). The phylogenetic tree shown in *SI Appendix*, Fig. S1*C* was constructed from this alignment using FastTree (52) and pruned to display only *E. coli* systems (Newick file for this tree in Dataset S10). EnvZ, RstB, and CpxA homologs from the ProGenomes2.0 database were identified and aligned using HMMER; specifically, jckhmmer was used to iteratively search the database for matches to the three kinase domain sequences. A phylogenetic tree was constructed using FastTree, and orthologs were classified based on clade identity. The resulting collection of sequences was further filtered by reciprocal HMMER to confirm that the best hit for each sequence in the *E. coli* genome was the correct paralog out of EnvZ, RstB, and CpxA. Each sequence maintained its species ID, allowing species with or without the relevant paralogs to be identified. The same process allowed YehU, BarA, and PhoR homologs to be identified, aligned, and filtered (fasta files for ortholog alignments are in Datasets S11–S16). The species tree in *SI Appendix*, Fig. S1*B* and Fig. 5*B* was obtained by pruning the tree constructed in ref. 49 (Newick file in Dataset S17). Progenomes 2.0 species IDs for species tested in Fig. 5*E* are 155892 (Caulobacter), 1770053 (Burkholderia), 1797492 (Betaproteobacterium sp.), and 595494 (Tolumonas).

### Statistical Calculations.

Two-sided Kolmogorov–Smirnov tests were used to determine whether there was a significant difference in distribution between counts of substitutions that do or do not cross-talk in multiple sequence alignments (MSAs) of HK orthologs. Two-sided Fisher’s exact tests were used to determine whether there was a significant difference between presence/absence within the MSA of substitutions that do or do not cross-talk. Enrichment in *SI Appendix*, Fig. S8*H* is calculated as counts in the first category of species, with counts in the second category subtracted, after scaling for the number of species in each category.

$$\begin{array}{c} \mathrm{enrichment}=\mathrm{count}\left(\mathrm{species}\;\mathrm{without}\;\mathrm{RstBA}\;\mathrm{or}\;\mathrm{CpxAR}\right)\\ \;-\mathrm{count}\left(\mathrm{species}\;\mathrm{with}\;\mathrm{RstBA}\;\mathrm{and}\;\mathrm{CpxAR}\right)\\ \;\times \frac{\#\mathrm{species}\;\mathrm{without}\;\mathrm{RstBA}\;\mathrm{or}\;\mathrm{CpxAR}}{\#\mathrm{species}\;\mathrm{with}\;\mathrm{RstBA}\;\mathrm{and}\;\mathrm{CpxAR}}. \end{array}$$

## Supplementary Material

Appendix 01 (PDF)

Dataset S01 (TXT)

Dataset S02 (TXT)

Dataset S03 (TXT)

Dataset S04 (TXT)

Dataset S05 (TXT)

Dataset S06 (XLSX)

Dataset S07 (CSV)

Dataset S08 (CSV)

Dataset S09 (CSV)

Dataset S10 (TXT)

Dataset S11 (TXT)

Dataset S12 (TXT)

Dataset S13 (TXT)

Dataset S14 (TXT)

Dataset S15 (TXT)

Dataset S16 (TXT)

Dataset S17 (TXT)

## Data Availability

Python scripts for analysis are available at https://github.com/d-ghose/laub (53). Datasets generated during this study have been deposited in the National Center for Biotechnology Information Sequence Read Archive (NCBI SRA). Raw reads can be found under BioProject ID PRJNA902002 (54). All other data are included in the manuscript and/or *SI Appendix*.
