# Supplementary material for: Marginal specificity in protein interactions constrains evolution of a paralogous family
Source: Proc Natl Acad Sci U S A. 2023 Apr 25;120(18):e2221163120. doi: 10.1073/pnas.2221163120 (PMC10160972; doi:10.1073/pnas.2221163120)
Supplement: Supplementary file 1 — Appendix 01 (PDF) [file pnas.2221163120.sapp.pdf]

PNAS

Supporting Information for
Marginal specificity in protein interactions constrains evolution of a paralogous family

Dia A. Ghose[1], Kaitlyn E. Przydzial[1], Emily M. Mahoney[1], Amy E. Keating[1,2,3], Michael T. Laub[1,4,5]

[1]Department of Biology, Massachusetts Institute of Technology, Cambridge, MA 02139, USA
[2]Department of Biological Engineering, Massachusetts Institute of Technology, Cambridge, MA 02139, USA
[3]Koch Center for Integrative Cancer Research, Massachusetts Institute of Technology, Cambridge, MA 02139, USA
[4]Howard Hughes Medical Institute, Massachusetts Institute of Technology, Cambridge, MA 02139, USA
[5]correspondence: laub@mit.edu, 617-324-0418

This PDF file includes:

Figures S1 to S8
Tables S1 to S2
Legends for Datasets S1 to S17

Other supporting materials for this manuscript include the following:

Datasets S1 to S17

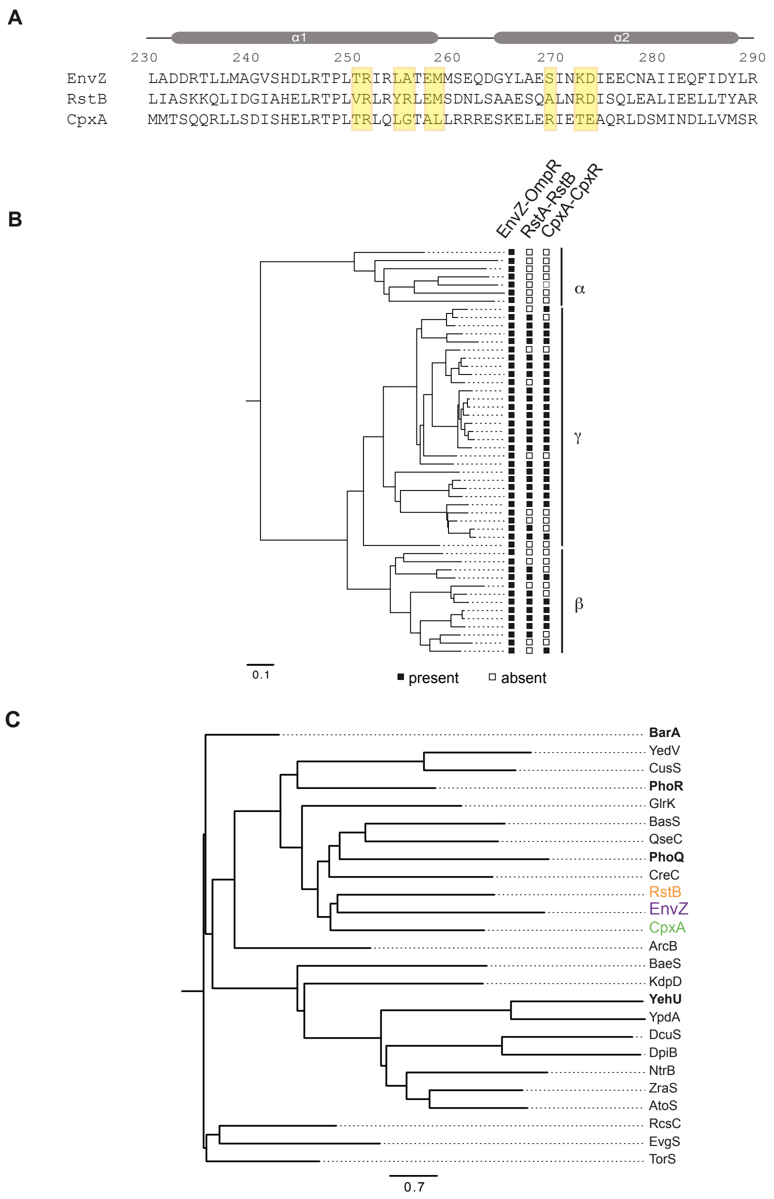

Fig. S1. Phylogenetic analysis of E. coli two-component signaling systems.

(A) Alignment of DHp domain sequences from EnvZ, RstB, and CpxA. Residues that strongly coevolve in HK and RR proteins are highlighted in yellow.

(B) Phylogenetic tree of α-, β-, and γ-proteobacteria, where closed and open squares indicate presence and absence, respectively, of EnvZ-OmpR, RstBA, and CpxAR. Scale bar indicates substitutions per site.

(C) Tree of *E. coli* histidine kinases with systems investigated in this study in larger text and color. Kinases in bold are controls used for comparison in the study. Scale bar indicates substitutions per site.

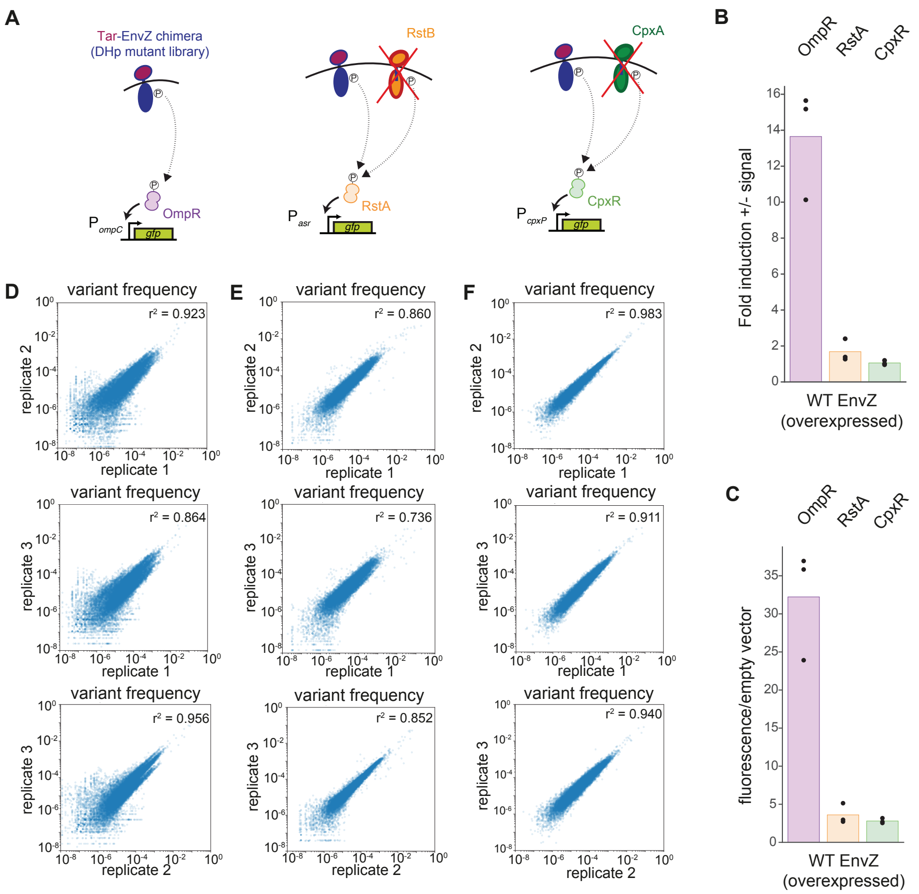

G

	Bin 1	Bin 2	Bin 3	Bin 4	Bin 5	Bin 6	Bin 7	Bin 8
Replicate 1 OFF	707	354	504	578	637	1002	1313	100
Replicate 2 OFF	526	355	467	422	412	805	533	20
Replicate 3 OFF	451	301	401	362	334	622	547	25
Replicate 1 ON	519	197	160	103	151	909	1221	100
Replicate 2 ON	526	195	159	100	101	595	1011	105
Replicate 3 ON	518	195	157	100	112	646	1071	100

H

	Bin 1	Bin 2	Bin 3	Bin 4	Bin 5	Bin 6	Bin 7	Bin 8
Replicate 1 OFF	256	992	1120	921	2408	1626	912	141
Replicate 2 OFF	151	638	1144	846	1150	1017	737	201
Replicate 3 OFF	136	568	1190	868	1115	920	612	148
Replicate 1 ON	268	1221	2059	1517	1039	830	579	131
Replicate 2 ON	202	814	1797	1302	1069	979	729	202
Replicate 3 ON	143	515	1165	838	1069	979	729	202

I

	Bin 1	Bin 2	Bin 3	Bin 4	Bin 5	Bin 6	Bin 7	Bin 8
Replicate 1 OFF	569	898	663	538	1595	876	287	122
Replicate 2 OFF	547	891	685	557	1558	878	297	124
Replicate 3 OFF	944	1056	717	526	1873	789	210	100
Replicate 1 ON	803	874	594	511	2462	882	215	100
Replicate 2 ON	601	827	599	530	1840	950	288	120
Replicate 3 ON	629	902	642	556	1861	959	290	120

J

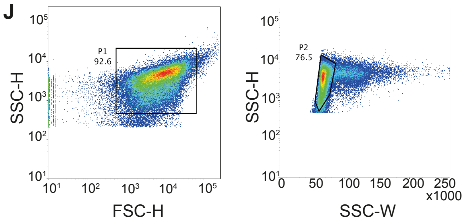

Fig. S2. Details of Sort-seq method and summary statistics.

(A) Summary of the three reporter strains used: OmpR reporter plasmid in Δ*envZ* strain, RstA reporter plasmid in Δ*envZ* Δ*rstB* Δ*acka-pta* strain, CpxR reporter plasmid in Δ*envZ* Δ*cpxA* Δ*acka-pta* strain. Cognate HKs of RstA and CpxR (RstB and CpxA) were removed from their reporter strains to prevent phosphorylation or dephosphorylation by these kinases from affecting the readout.

(B) Fold induction (+/- signal) of each reporter with wild-type EnvZ expressed at the level used in the library. n=3 biological replicates.

(C) Fluorescence levels (+ signal) of each reporter with wild-type EnvZ expressed at the level used in the library. Levels were normalized to an empty vector control for each strain. n=3 biological replicates.
(D-F) Scatter plots displaying the correlations between the bin frequencies of individual variants measured in independent replicates for the OmpR (D), RstA (E), and CpxR (F) reporters.
(G-I) Counts of cells sorted into each bin, in thousands, for each replicate and condition, for the OmpR (G), RstA (H), and CpxR (I) reporters.
(J) FACS gating strategy for isolating live single cells. SSC-H/W = Side Scatter Pulse Height/Width, FSC-H = Forward Scatter Pulse-Height.

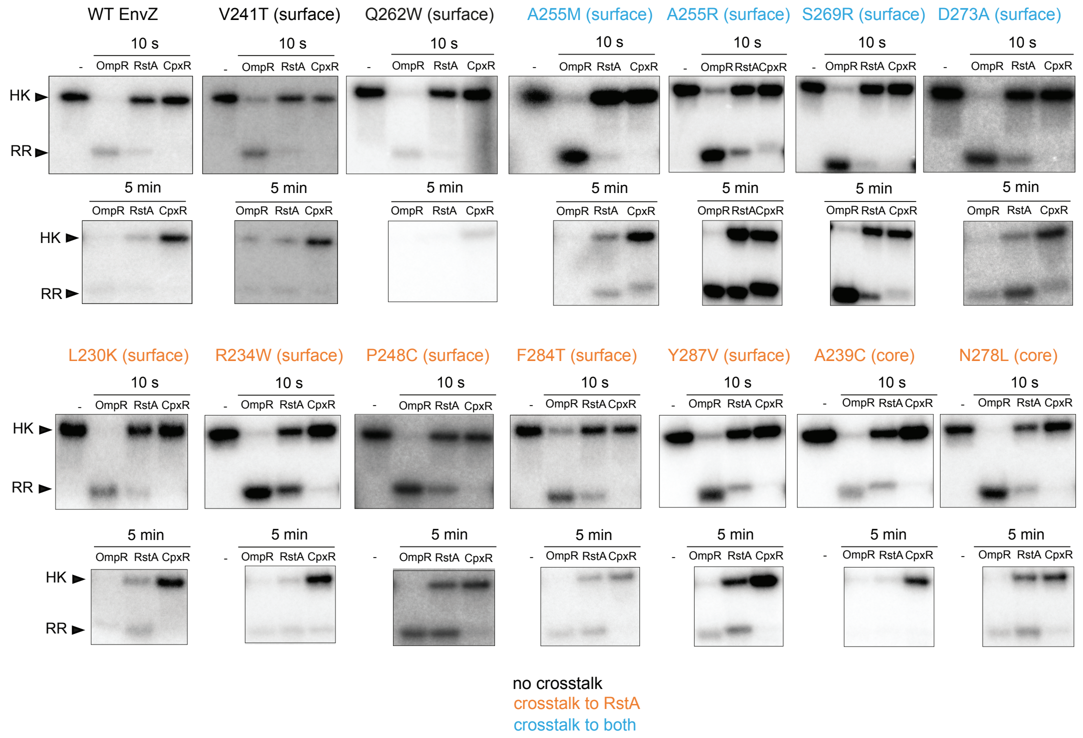

Fig. S3. In vitro biochemical validation of high-throughput screen results. Phosphotransfer assays conducted with a range of EnvZ variants against OmpR, RstA, and CpxR at 10 s and 5 min time points. The upper band shows ^{32}P-ATP incorporated into the autophosphorylated kinase, which depletes upon phosphotransfer to the response regulator. The lower band shows ^{32}P-ATP incorporated into the response regulator, which at first accumulates upon transfer, and then depletes due to phosphatase activity by the kinase. Color of the text listing a given substitution indicates cross-talk activity seen in the deep mutational scanning, with black, orange, and blue indicating no cross-talk, cross-talk to RstA, and cross-talk to both, respectively. Cross-talk is defined as 5-fold increases in fluorescence relative to WT EnvZ. Wild-type EnvZ and two variants that showed no cross-talk activity in the screen are shown, along with 4 substitutions at coevolving residues that showed cross-talk to RstA and CpxR, and 7 substitutions at distal positions that showed cross-talk to RstA. Residues on the surface or within the core of the DHp domain are indicated. The Q262W variant shows depletion of autophosphorylated kinase with all three RRs, and F284T shows some depletion with CpxR even though they were not found to cross-talk in the screen. This may be because they showed low levels of activity which were not able to be separated from WT EnvZ by sorting or did not meet the 5-fold threshold to be considered cross-talk.

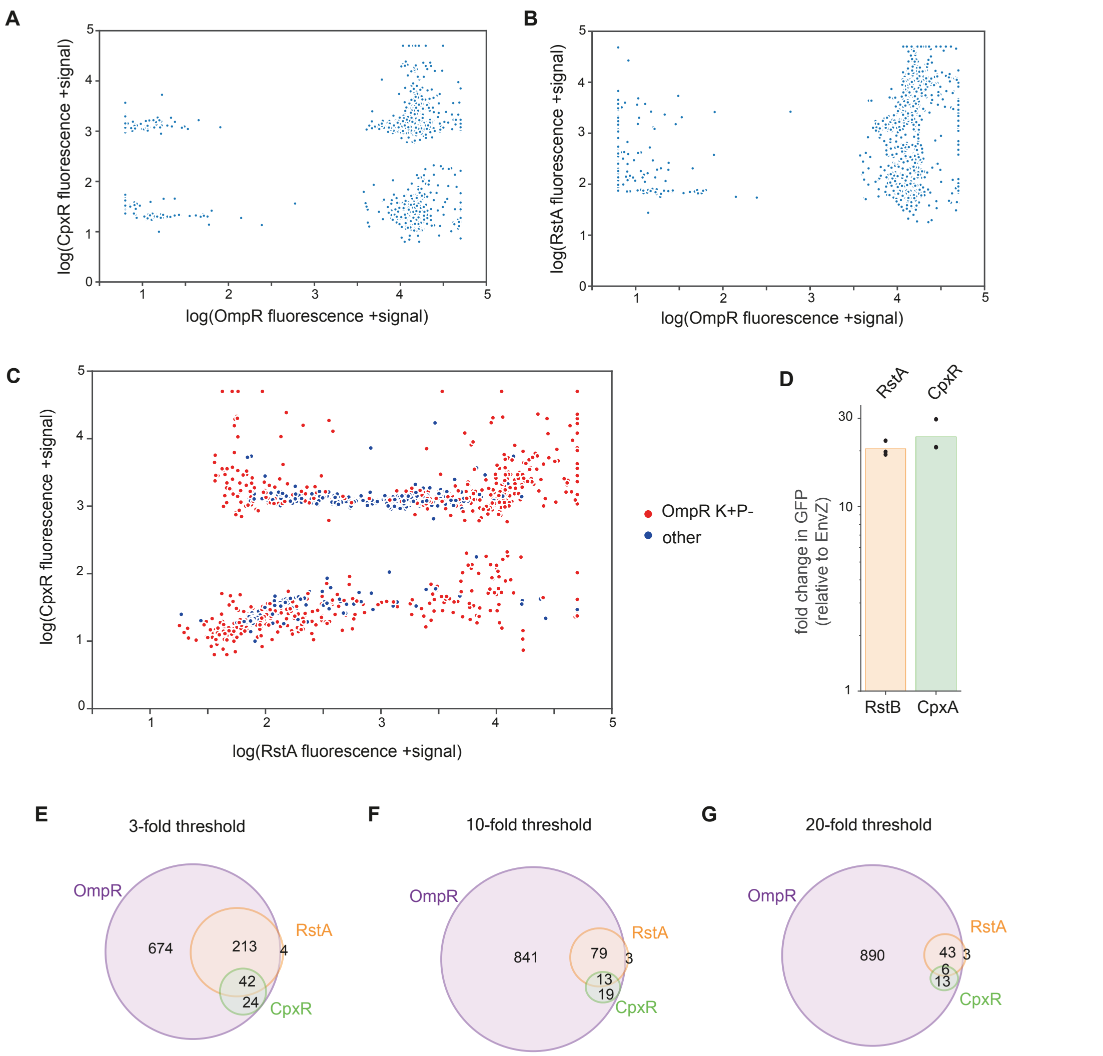

Fig. S4. Validation of mutation effect sizes and thresholds.

(A-B) Scatter plot displaying the correlations between fluorescence values of EnvZ variants screened against the (A) CpxR and OmpR reporters or (B) RstA and OmpR reporters in +signal condition.

(C) Scatter plot displaying the correlations between +signal fluorescence values of EnvZ variants screened against CpxR vs. RstA. Red dots show variants that retain kinase activity but not phosphatase activity towards OmpR (K+P- variants). There is little correlation between the fluorescence values of variants with the three different reporters, showing that mutation effects are largely specific to each RR, and not caused by general properties such as expression level or kinase activity. There is also no relationship between loss of phosphatase activity towards OmpR and mutation effect towards RstA and CpxR, demonstrating that simply perturbing the equilibrium of kinase/phosphatase activity towards kinase is not sufficient to generate cross-talk.

(D) Fold change in fluorescence of RstA and CpxR reporters relative to wild-type EnvZ when tested with chimeric HKs containing the Tar sensor domain and RstB or CpxA cytoplasmic domains, respectively. n=3 biological replicates.

(E-G) Overlap of single-substitution EnvZ variants with activity towards different RRs. The OmpR set contains sequences which showed kinase activity towards OmpR at a comparable level to wild-type EnvZ (within 5-fold). The RstA and CpxR sets contain sequences which showed (E) ≥ 3-fold, (F) ≥ 10-fold, or (G) ≥ 20-fold increases in activity towards RstA or CpxR relative to wild-type EnvZ.

A

% cross-talking substitutions	RstA	CpxR
Total	25	5
RstB/CpxA-matching	45	9

B

model 1

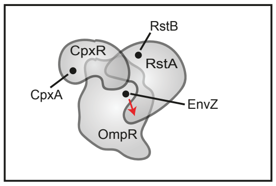

substitutions that do not resemble RstB can cause cross-talk to RstA

model 2

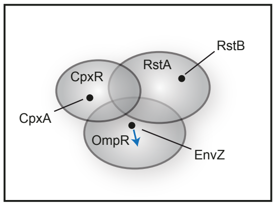

substitutions that do not resemble RstB do not cause cross-talk to RstA

Fig. S5. Niches in sequence space are irregularly shaped.

(A) Table showing percentage of functional sequences (that have kinase activity for at least one regulator) that cross-talk to RstA or CpxR, among all functional sequences (top row) or among functional sequences that have a substitution corresponding to the residue found at the equivalent position in the cognate HK of RstA or CpxR (bottom row).

(B) Sequence space diagram illustrating two possible models for the shapes of two-component signaling protein niches. In model 1, niches are irregularly shaped and changing the EnvZ sequence to be more similar to RstB or CpxA is not the only way to generate cross-talk towards RstA or CpxR. In model 2, niches are regularly shaped and substitutions that do not resemble the cognate kinases of RstA or CpxR do not generate cross-talk.

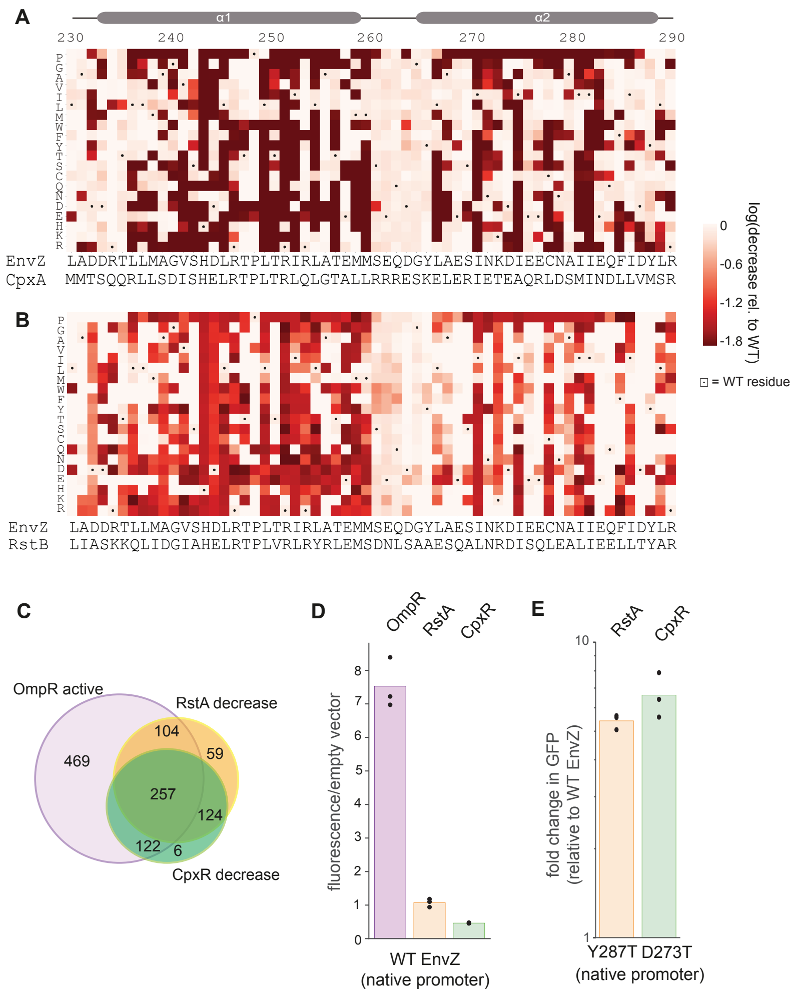

Fig. S6. Many substitutions reduce interactions with non-cognate RRs.

(A-B) Heatmaps of (A) CpxR or (B) RstA reporter data where the values represent $\log_{10}$(fluorescence) of variants in +signal condition. Wild-type EnvZ is set to 0, and color-coded as white, and is the maximum value shown (red shows decreases). All variants with increased fluorescence are shown as 0. Dots mark wild-type EnvZ residues.

(C) Overlap of single-substitution EnvZ variants with activity towards different RRs: the OmpR set contains sequences that show kinase activity towards OmpR at a comparable level to wild-type EnvZ (within 5-fold). RstA and CpxR sets contain sequences with ≥ 5-fold decreases in activity towards RstA or CpxR relative to wild-type EnvZ.

(D) Fluorescence levels for OmpR, RstA, and CpxR reporters following expression of wild-type EnvZ under its native promoter, normalized to levels seen with an empty vector for each strain. n=3 biological replicates.

(E) Variants that show 5-fold cross-talk to RstA or CpxR in the Sort-seq screen (where kinase variants are overexpressed) still show cross-talk when expressed from the native promoter. n=3 biological replicates.

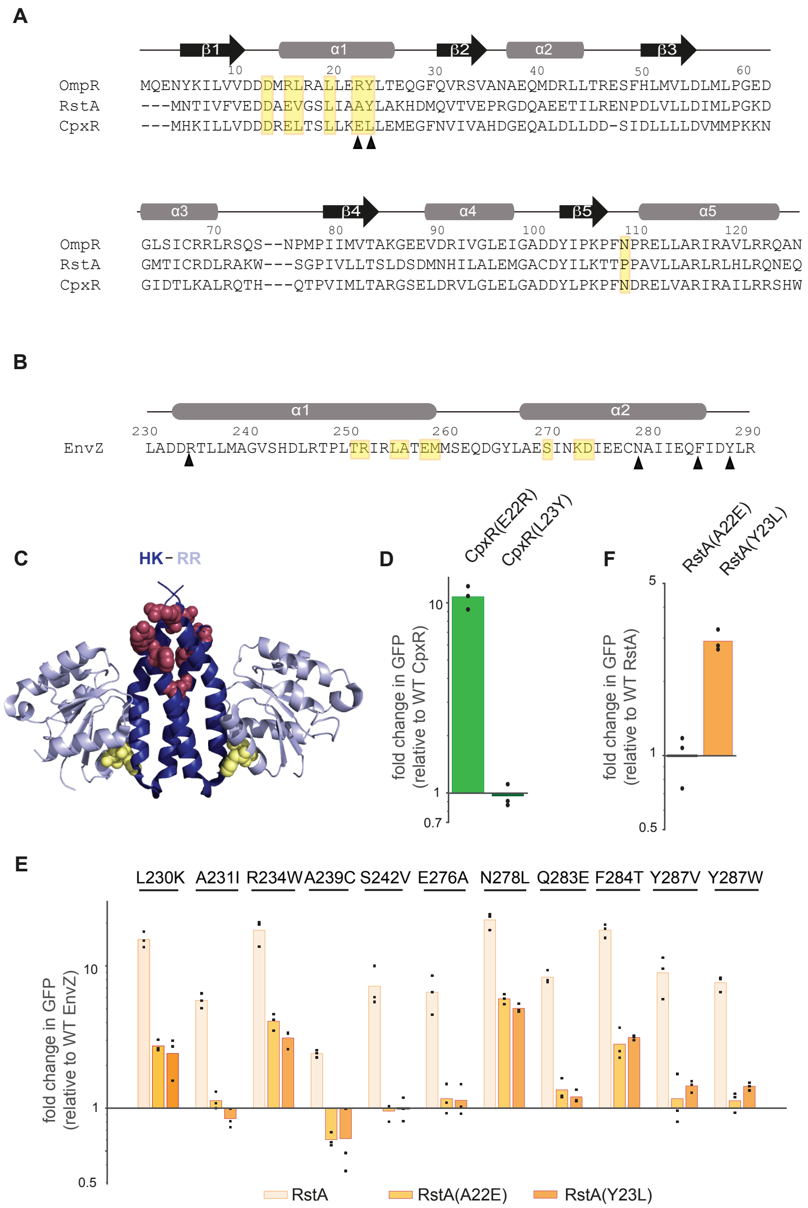

Fig. S7. Additional characterization of RR substitutions.

(A) Alignment of OmpR, RstA, and CpxR with coevolving residues highlighted in yellow. Arrowheads mark the two coevolving positions that differ significantly between CpxR and both OmpR and RstA.

(B) EnvZ DHp domain sequence. Coevolving residues are highlighted in yellow. Arrowheads mark positions on EnvZ where secondary interface substitutions lead to cross-talk to RstA and that were tested for epistasis with RR substitutions.

(C) Model of HK-RR complex with HK in deep blue and RR in light blue. Yellow spheres on RR indicate coevolving residues of RstA and CpxR that were substituted and red spheres on HK indicate distal residues of EnvZ that were substituted and tested against the variant RRs.

(D) Fold changes in fluorescence of CpxR(E22R) and CpxR(L23Y) with wild-type EnvZ, relative to wild-type CpxR with wild-type EnvZ. n=3 biological replicates.

(E) Fold changes in fluorescence relative to wild-type EnvZ for 7 additional distal single substitutions in EnvZ, against wild-type RstA, RstA(A22E), and RstA(Y23L). Data for 4 substitutions shown in Fig 4h are replicated here. n=3 biological replicates.

(F) Fold changes in fluorescence of RstA(A22E) and RstA(Y23L) with wild-type EnvZ, relative to wild-type RstA with wild-type EnvZ. n=3 biological replicates. When compared to wild-type RstA, the substitutions tested in RstA do not lead to less phosphotransfer from wild-type EnvZ, suggesting that the decrease in activation by the EnvZ variants is not due to protein misfolding or lower expression.

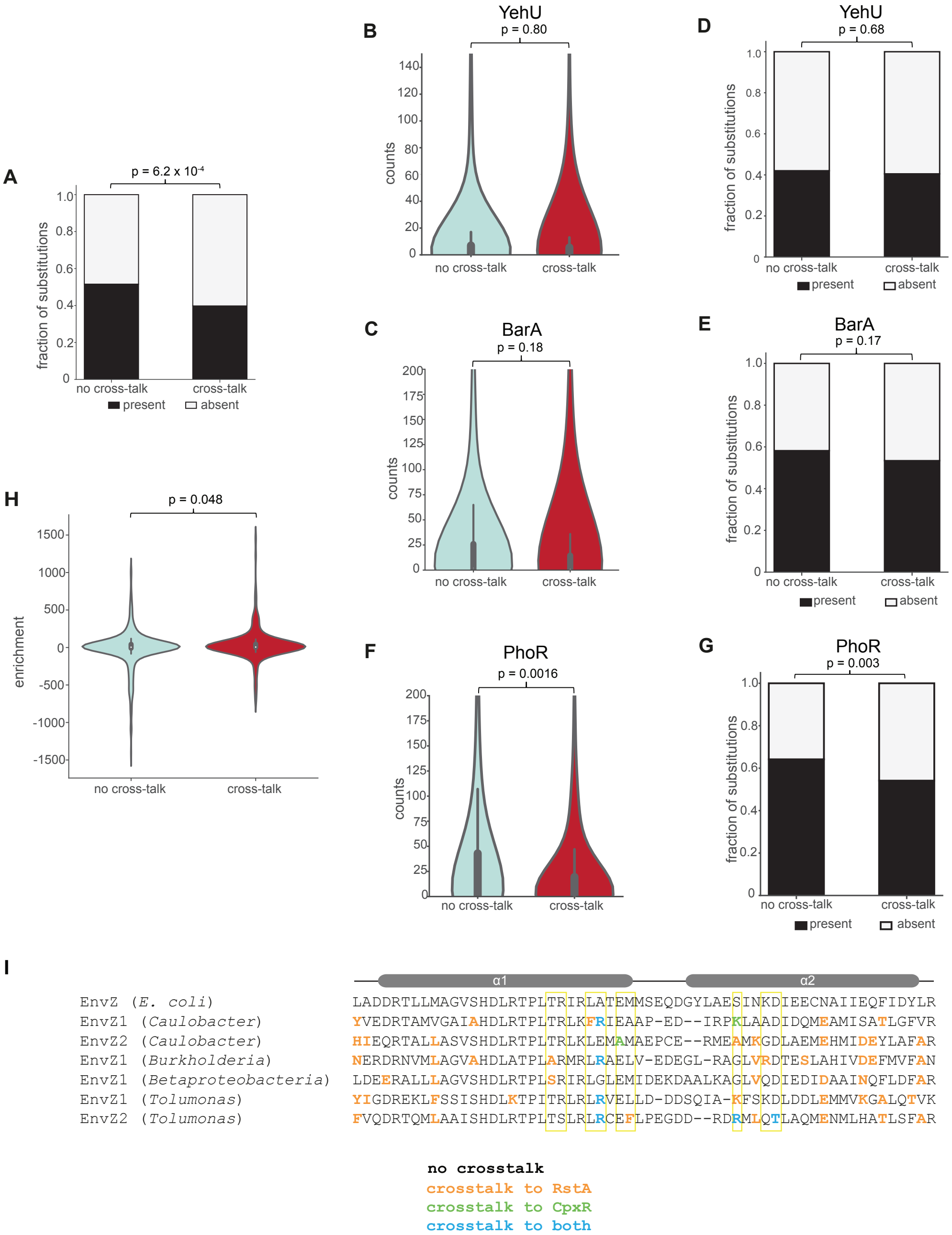

Fig. S8. Additional bioinformatic analyses of histidine kinase ortholog sequences.

(A) Fraction of substitutions observed to cross-talk or not (see Fig. 3) that are present or absent within the multiple-sequence alignment of 1019 EnvZ orthologs ($p = 6.2 \times 10^{-4}$, Fisher's exact test).

(B) Violin plots show the distributions of counts of single substitutions found at the equivalent position in 594 YehU orthologs. Counts are shown for two categories of substitution: those which do produce cross-talk to either RstA or CpxR, and those which do not (see Fig. 3, p = 0.80, Kolmogorov-Smirnov test), The inner box shows the quartiles and the whiskers show the range except for outliers.

(C) Same as (B) but for 1,088 BarA orthologs (p = 0.18, Kolmogorov-Smirnov test).

(D) Same as (A) but for YehU (p = 0.67, Fisher's exact test).

(E) Same as (A) but for BarA (p = 0.17, Fisher's exact test).

(F) Same as (B) but for 1,067 PhoR orthologs (p = 0.0016, Kolmogorov-Smirnov test).

(G) Same as (A) but for PhoR (p = 0.0030, Fisher's exact test).
(H) Violin plot showing distributions of enrichment of substitutions that either do or do not cross-talk in species that have lost RstBA and CpxAR, relative to species that retain RstBA and CpxAR (see Methods for enrichment calculation, p = 0.048, Kolmogorov-Smirnov test).
(I) Alignment of EnvZ orthologs from species with duplications that have also lost RstBA and CpxAR, showing that these proteins have residues that cause cross-talk in the *E. coli* EnvZ background, as measured in our screen. Positions boxed in yellow are coevolving residues.

Table S1. Strain table

Name	Base strain	Genotype	Plasmids
**ML1803**	Yale BW28357	Δ*envZ*	
**ML3963**	Yale BW28357	Δ*envZ* Δ*ackA-pta* Δ*rstB*	
**ML3964**	Yale BW28357	Δ*envZ* Δ*ackA-pta* Δ*cpxA*	
**ML3965**	Yale BW28357	Δ*envZ* Δ*ackA-pta* Δ*phoQ*	
**ML3966**	Yale BW28357	Δ*envZ*	pOmpR
**ML3967**	Yale BW28357	Δ*envZ* Δ*ackA-pta* Δ*rstB*	pRstA
**ML3968**	Yale BW28357	Δ*envZ* Δ*ackA-pta* Δ*cpxA*	pCpxR
**ML3969**	Yale BW28357	Δ*envZ* Δ*ackA-pta* Δ*phoQ*	pPhoP
**ML3970**	Yale BW28357	Δ*envZ* Δ*ackA-pta* Δ*cpxA cpxR::cpxR E22R*	pCpxR
**ML3971**	Yale BW28357	Δ*envZ* Δ*ackA-pta* Δ*cpxA cpxR::cpxR L23Y*	pCpxR
**ML3972**	Yale BW28357	Δ*envZ* Δ*ackA-pta* Δ*rstB rstA::rstA A22E*	pRstA
**ML3973**	Yale BW28357	Δ*envZ* Δ*ackA-pta* Δ*rstB rstA::rstA Y23L*	pRstA
**ML3974**	DH5α		pDG116
**ML3975**	DH5α		pOmpR
**ML3976**	DH5α		pRstA
**ML3977**	DH5α		pCpxR
**ML3978**	DH5α		pPhoP
**ML3979**	DH5α		pDG169
**ML3980**	DH5α		pDG170
**ML3981**	DH5α		pDG220
**ML3982**	DH5α		pDG222
**ML3983**	DH5α		pDG223
**ML3984**	DH5α		pKP33
**ML3985**	DH5α		pDG164
**ML3986**	DH5α		pDG165

**ML3987**	DH5α		pDG166
**ML3988**	DH5α		pDG167
**ML3989**	DH5α		pDG273
**ML3990**	DH5α		pDG274
**ML3991**	DH5α		pDG071
**ML3992**	DH5α		pDG119
**ML3993**	DH5α		pDG120
**ML3994**	DH5α		pDG121
**ML3995**	DH5α		pDG122
**ML3996**	DH5α		pDG128
**ML3997**	DH5α		pDG129
**ML3998**	DH5α		pDG130
**ML3999**	DH5α		pDG133
**ML4000**	DH5α		pDG137
**ML4001**	DH5α		pDG138
**ML4002**	DH5α		pDG156
**ML4003**	DH5α		pDG157
**ML4004**	DH5α		pDG158
**ML4005**	DH5α		pDG159
**ML4006**	DH5α		pDG160
**ML4007**	DH5α		pDG161
**ML4008**	DH5α		pDG162
**ML4009**	DH5α		pDG163
**ML4010**	DH5α		pDG171
**ML4011**	DH5α		pDG172
**ML4012**	DH5α		pDG173
**ML4013**	DH5α		pDG174
**ML4014**	DH5α		pDG192
**ML4015**	DH5α		pDG193
**ML4016**	DH5α		pDG194
**ML4017**	DH5α		pDG197
**ML4018**	DH5α		pDG198
**ML4019**	DH5α		pDG199
**ML4020**	DH5α		pDG200
**ML4021**	DH5α		pDG201
**ML4022**	DH5α		pDG202
**ML4023**	DH5α		pDG203
**ML4024**	DH5α		pDG182
**ML4025**	DH5α		pDG183
**ML4026**	DH5α		pDG184
**ML4027**	DH5α		pDG185

**ML4028**	DH5α		pDG186
**ML4029**	DH5α		pDG187
**ML4030**	DH5α		pDG204
**ML4031**	DH5α		pDG205
**ML4032**	DH5α		pDG206
**ML4033**	DH5α		pDG207
**ML4034**	DH5α		pDG208
**ML4035**	DH5α		pDG264
**ML4036**	DH5α		pDG265
**ML4037**	DH5α		pDG263
**ML4038**	DH5α		pDG276
**ML4039**	TOP10		Taz single mutant library (pDG116 background)
**ML4040**	Yale BW28357	Δ*envZ*	pOmpR + Taz single mutant library
**ML4041**	Yale BW28357	Δ*envZ* Δ*ackA-pta* Δ*rstB*	pRstA + Taz single mutant library
**ML4042**	Yale BW28357	Δ*envZ* Δ*ackA-pta* Δ*cpxA*	pCpxR + Taz single mutant library
**ML4043**	Yale BW28357	Δ*envZ* Δ*ackA-pta* Δ*phoQ*	pPhoP + Taz single mutant library

Table S2. Plasmid table

**Name**	**Description**
**pDG116**	P_{lpp}-*taz*, specR, pSC101
**pOmpR**	P_{ompC}-*gfp*, cmR, p15a
**pRstA**	P_{asr}-*gfp*, cmR, p15a
**pCpxR**	P_{cpxP}-*gfp*, cmR, p15a
**pPhoP**	P_{mgrB}-*gfp*, cmR, p15a
**pDG169**	P_{lpp}-*taz(Caulobacter EnvZ1)*, specR, pSC101
**pDG170**	P_{lpp}-*taz(Caulobacter EnvZ2)*, specR, pSC101
**pDG220**	P_{lpp}-*taz(Burkholderia EnvZ1)*, specR, pSC101
**pDG222**	P_{lpp}-*taz(Tolumonas EnvZ1)*, specR, pSC101
**pDG223**	P_{lpp}-*taz(Tolumonas EnvZ2)*, specR, pSC101
**pKP33**	P_{lpp}-*taz(Betaproteobacteria EnvZ1)*, specR, pSC101
**pDG164**	Pt7-6His-MBP-EnvZ, ampR, ColEI
**pDG165**	Pt7-6His-Trx-OmpR, ampR, ColEI
**pDG166**	Pt7-6His-Trx-RstA, ampR, ColEI
**pDG167**	Pt7-6His-Trx-CpxR, ampR, ColEI
**pDG273**	P_{lpp}-*tar-RstB*, specR, pSC101
**pDG274**	P_{lpp}-*tar-CpxA*, specR, pSC101
**pDG071**	P_{ompR3}-*envZ*, specR, pSC101
**pDG119**	P_{lpp}-*taz M258L*, specR, pSC101
**pDG120**	P_{lpp}-*taz S269R*, specR, pSC101
**pDG121**	P_{lpp}-*taz D273E*, specR, pSC101
**pDG122**	P_{lpp}-*taz A255R*, specR, pSC101
**pDG128**	P_{lpp}-*taz H243A*, specR, pSC101
**pDG129**	P_{lpp}-*taz V241G*, specR, pSC101
**pDG130**	P_{lpp}-*taz A239T*, specR, pSC101
**pDG133**	P_{lpp}-*taz E257A*, specR, pSC101
**pDG137**	P_{lpp}-*taz T250V*, specR, pSC101
**pDG138**	P_{lpp}-*taz L254Y*, specR, pSC101
**pDG156**	P_{lpp}-*taz P248C*, specR, pSC101
**pDG157**	P_{lpp}-*taz D273A*, specR, pSC101
**pDG158**	P_{lpp}-*taz V241T*, specR, pSC101
**pDG159**	P_{lpp}-*taz F284T*, specR, pSC101
**pDG160**	P_{lpp}-*taz A255M*, specR, pSC101
**pDG161**	P_{lpp}-*taz Q262W*, specR, pSC101
**pDG162**	P_{lpp}-*taz M258F*, specR, pSC101
**pDG163**	P_{lpp}-*taz L249K*, specR, pSC101
**pDG171**	P_{lpp}-*taz L249W*, specR, pSC101
**pDG172**	P_{lpp}-*taz R253Y*, specR, pSC101
**pDG173**	P_{lpp}-*taz I269Y*, specR, pSC101

**pDG174**	P_{lpp}-*taz D286V*, specR, pSC101
**pDG192**	P_{lpp}-*taz A231I*, specR, pSC101
**pDG193**	P_{lpp}-*taz Y287W*, specR, pSC101
**pDG194**	P_{lpp}-*taz Q283E*, specR, pSC101
**pDG197**	P_{lpp}-*taz S242V*, specR, pSC101
**pDG198**	P_{lpp}-*taz E276A*, specR, pSC101
**pDG199**	P_{lpp}-*taz L230K*, specR, pSC101
**pDG200**	P_{lpp}-*taz R234W*, specR, pSC101
**pDG201**	P_{lpp}-*taz N278L*, specR, pSC101
**pDG202**	P_{lpp}-*taz Y282V*, specR, pSC101
**pDG203**	P_{lpp}-*taz A239C*, specR, pSC101
**pDG182**	Pt7-6His-MBP-EnvZ P248C, ampR, ColEI
**pDG183**	Pt7-6His-MBP-EnvZ D273A, ampR, ColEI
**pDG184**	Pt7-6His-MBP-EnvZ V241T, ampR, ColEI
**pDG185**	Pt7-6His-MBP-EnvZ F284T, ampR, ColEI
**pDG186**	Pt7-6His-MBP-EnvZ A255M, ampR, ColEI
**pDG187**	Pt7-6His-MBP-EnvZ Q262W, ampR, ColEI
**pDG204**	Pt7-6His-MBP-EnvZ L230K, ampR, ColEI
**pDG205**	Pt7-6His-MBP-EnvZ R234W, ampR, ColEI
**pDG206**	Pt7-6His-MBP-EnvZ N278L, ampR, ColEI
**pDG207**	Pt7-6His-MBP-EnvZ Y287V, ampR, ColEI
**pDG208**	Pt7-6His-MBP-EnvZ A239C, ampR, ColEI
**pDG264**	Pt7-6His-MBP-EnvZ A255R, ampR, ColEI
**pDG265**	Pt7-6His-MBP-EnvZ S264R, ampR, ColEI
**pDG263**	P_{ompR3}-*envZ Y287T*, specR, pSC101
**pDG276**	P_{ompR3}-*envZ D273T*, specR, pSC101

Dataset S1 (separate file). Plasmid sequence file for OmpR *gfp* reporter.

Dataset S2 (separate file). Plasmid sequence file for RstA *gfp* reporter.

Dataset S3 (separate file). Plasmid sequence file for CpxR *gfp* reporter.

Dataset S4 (separate file). Plasmid sequence file for PhoP *gfp* reporter.

Dataset S5 (separate file). Plasmid sequence file for pDG116 (*taz* expression construct).

Dataset S6 (separate file). Primer table.

Dataset S7 (separate file). Fitted Gaussian means and standard deviations for each variant for OmpR reporter.

Dataset S8 (separate file). Fitted Gaussian means and standard deviations for each variant for RstA reporter.

Dataset S9 (separate file). Fitted Gaussian means and standard deviations for each variant for CpxR reporter.

Dataset S10 (separate file). Newick file for phylogenetic tree of *E. coli* histidine kinases shown in Fig. S1C.

Dataset S11 (separate file). Fasta file for alignment of EnvZ orthologs.

Dataset S12 (separate file). Fasta file for alignment of RstB orthologs.

Dataset S13 (separate file). Fasta file for alignment of CpxA orthologs.

Dataset S14 (separate file). Fasta file for alignment of YehU orthologs.

Dataset S15 (separate file). Fasta file for alignment of BarA orthologs.

Dataset S16 (separate file). Fasta file for alignment of PhoR orthologs.

Dataset S17 (separate file). Newick file for phylogenetic tree of proteobacterial species shown in Fig. S1B and Fig. 5B.
